# Synthesis of homo- and heteromultivalent carbohydrate-functionalized oligo(amidoamines) using novel glyco-building blocks

**DOI:** 10.3762/bjoc.9.276

**Published:** 2013-11-07

**Authors:** Felix Wojcik, Sinaida Lel, Alexander G O’Brien, Peter H Seeberger, Laura Hartmann

**Affiliations:** 1 Department of Biomolecular Systems, Max Planck Institute of Colloids and Interfaces, Research Campus Golm, 14424 Potsdam, Germany, and Institute for Chemistry and Biochemistry, Freie Universität Berlin, Arnimallee 22, 14195 Berlin, Germany

**Keywords:** continuous flow, flow chemistry: flow synthesis, glycoligands, multivalency, photochemistry, solid-phase synthesis, thiol–ene, thioglycosides

## Abstract

We present the solid phase synthesis of carbohydrate-functionalized oligo(amidoamines) with different functionalization patterns utilizing a novel alphabet of six differently glycosylated building blocks. Highly efficient in flow conjugation of thioglycosides to a double-bond presenting diethylentriamine precursor is the key step to prepare these building blocks suitable for fully automated solid-phase synthesis. Introduction of the sugar ligands via functionalized building blocks rather than postfunctionalization of the oligomeric backbone allows for the straightforward synthesis of multivalent glycoligands with full control over monomer sequence and functionalization pattern. We demonstrate the potential of this building-block approach by synthesizing oligomers with different numbers and spacing of carbohydrates and also show the feasibility of heteromultivalent glycosylation patterns by combining building blocks presenting different mono- and disaccharides.

## Introduction

Multivalent carbohydrate ligand–protein receptor interactions play a key role for many events in glycobiology such as cell–cell or pathogen recognition [1]. Therefore, carbohydrate functionalization of non-natural materials such as polymers or dendrimers allows for bioactive materials that are used to modulate cellular behavior [1–3]. Since single carbohydrate ligand–protein interactions are usually weak [4], several sugar ligands have to be introduced in order to achieve the desired biological effect [4]. This multivalent presentation of ligands then results in an increased binding affinity to the targeted protein receptors [4]. It is well understood that the number and spacing of ligands have a tremendous influence on the resulting binding and thus biological properties [5–7]. Therefore, in order to understand and explore these interactions and their potential for biomedical applications, a more detailed look at the binding mechanisms as well as structure–activity relationship studies of multivalent glycomaterials is required.

Multivalent sugar presentation has been realized on a variety of different scaffolds such as polymers [8–9], dendrimers [10] or naturally-occurring scaffolds such as peptides [11–13] or oligonucleotides [14]. Such constructs have contributed to our current understanding of multivalent interactions [5]. Nevertheless, binding studies on multivalent compounds with different scaffold architectures or combinations of different sugar ligands are limited due to the often undefined chemical composition, limited variations in architecture and functionalization as well as unspecific biological activity of the scaffolds. Precision oligo/polymers are a novel class of defined artificial scaffolds having the potential to bridge this current gap of artificial carbohydrate presenting scaffolds and to be an important platform for structure–activity relationship studies [15–17]. Precision macromolecules combine the advantages of synthetic scaffolds such as polymers with the advantages of naturally-occurring scaffolds such as peptides as they are highly defined, versatile in their structure (linear or branched) [18] and biocompatible with a decreased risk of inherent immunogenicity [19].

Recently, we showed that monodisperse, sequence-defined glycooligomers obtained by sequential addition of building blocks on solid support are valuable tools for tuning and understanding carbohydrate–lectin interactions [20]. Carbohydrate conjugation was achieved by copper-catalyzed azide alkyne cycloaddtion (CuAAC) of carbohydrate ligands on alkyne presenting oligomers [21]. As an alternative conjugation approach to CuAAc, a very efficient thiol–ene coupling (TEC) [22–25] protocol in a continuous flow photoreactor was developed involving post functionalization of alkene presenting oligomers by thioglycosides [26]. The flow system allows for precise control over the reaction conditions, is easy to scale up and provides efficient irradiation of the samples by virtue of a sub-millimeter path length. Continuous removal of the desired product minimizes unwanted secondary reaction pathways [27–39]. We also introduced the so-called building block approach in the context of thiol–ene coupling via the continuous-flow technique. A first example involved conjugating a glucose ligand to a building block and subsequent solid phase assembly of a glycooligomer [26].

**Figure 1:**
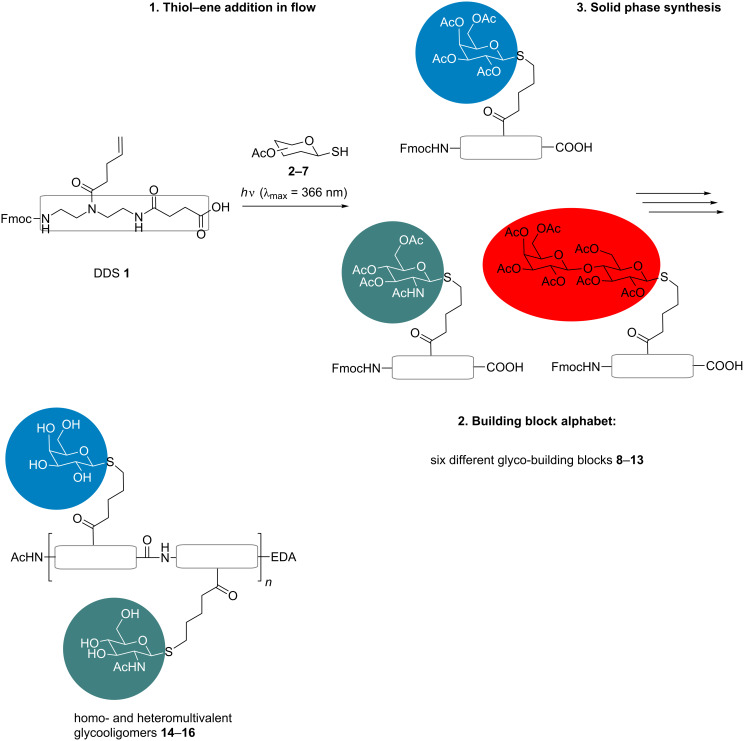
Versatile synthetic strategy for access to multivalent glycoligands: First, the building block DDS **1** is functionalized with different acetyl-protected thioglycosides **2**–**7** via thiol–ene addition in flow at >275 nm, resulting in a building block alphabet of six different glyco-building blocks **8**–**13**. These building blocks are then applied for the solid-phase synthesis of sequence-defined glycooligomers **14**–**16**.

When compared to postfunctionalization, the building-block approach allows not only control of the ligand positioning, but also enables well-defined sequences with different types of ligands: Simply by choosing from an alphabet of building blocks, applying them for solid-phase synthesis and final cleavage from the resin, the desired multivalent structures can be obtained. Heteromultivalent glycooligomers presenting different sugars at different positions along the scaffold should be accessible by combining different carbohydrate functionalized building blocks and without the requirement of complex protecting group or sequential functionalization strategies [40]. In order to explore the feasibility of the building-block approach for the synthesis of precision glycooligo/polymers, in this work we report on the reaction of several thioglycosides and the double bond presenting diethylenetriamine succinic acid building block (DDS) **1**, giving access to a small alphabet of carbohydrate-functionalized building blocks. TEC in flow enabled determining the reactivity of each thioglycoside at >275 nm, leading to optimized reaction conditions for the production of six glycosylated building blocks (Figure 1). These building blocks can then be used for the assembly of monodisperse, sequence-defined glycooligomers via fully automated standard amide coupling. Straightforward variations in the scaffold architecture, number and distance of sugar ligands as well as the sequence-defined introduction of different sugars are demonstrated by choosing different building block combinations during solid-phase synthesis.

## Results and Discussion

For the preparation of the desired sugar building blocks double-bond presenting building block DDS **1** and thioglycosides **2**–**7** are required. The large scale synthesis of DDS **1** was achieved according to a published procedure [26]. The required β-thioglycosides **2**–**7** were prepared via their corresponding glycosyl bromides followed by S_N_2 displacement of the anomeric bromide with thiourea [41] or Na_2_S/CS_2_ [42].

**Scheme 1:**
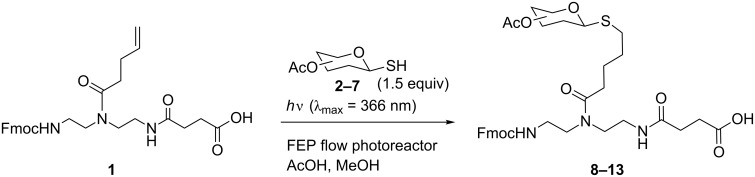
Carbohydrate functionalization of DDS.

**Figure 2:**
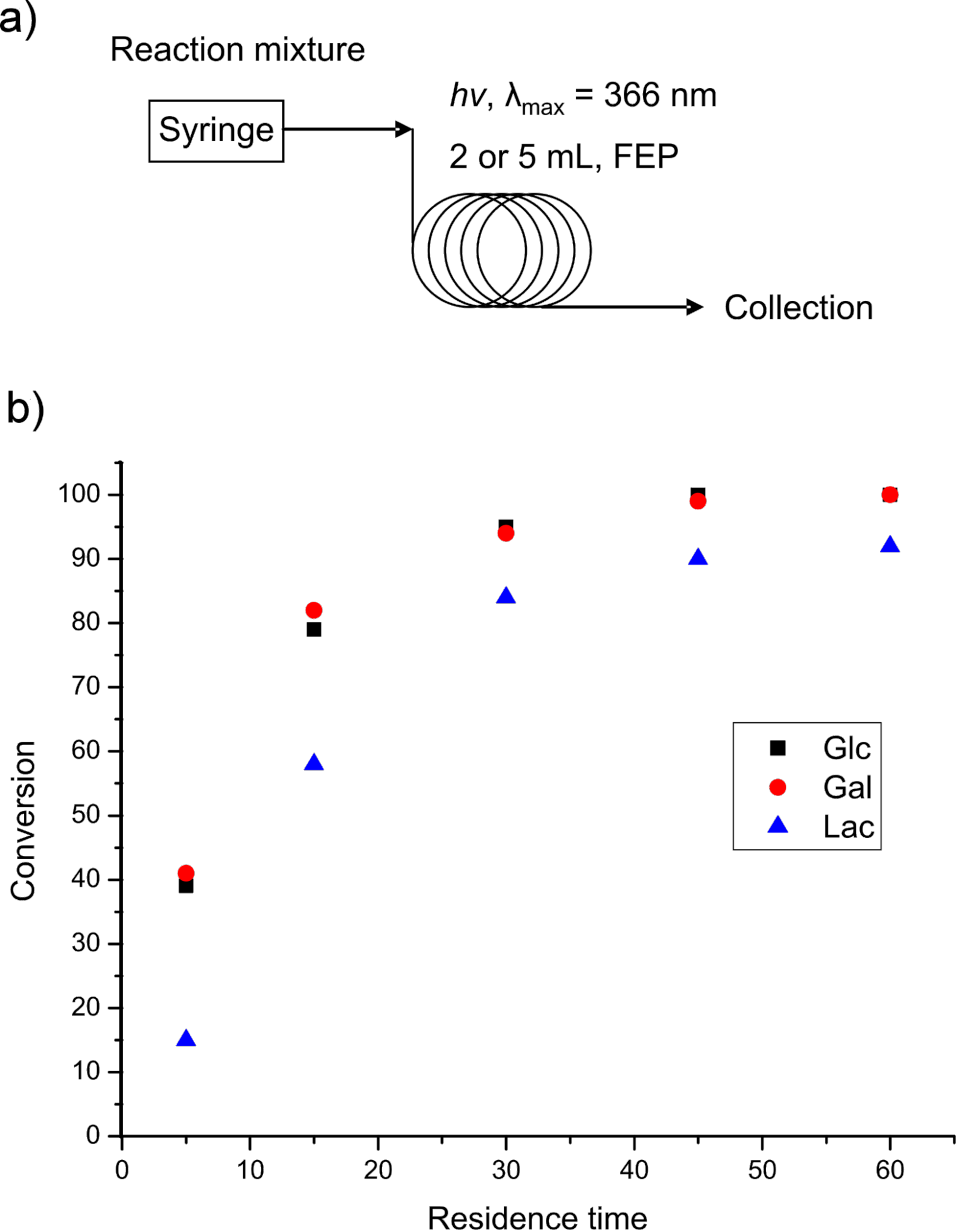
a) Schematic diagram of the TEC photoflow reactor. b) Plot of residence time versus conversion for the addition of the β-Glc(OAc)_4_-SH **2**, β-Gal(OAc)_4_-SH **3** and β-Lac(OAc)_7_-SH **7** to the DDS building block **1**.

DDS **1** and thioglycosides **2**–**7** were subjected to TEC in flow at >275 nm (Scheme 1). A FEP flow photoreactor equipped with a Pyrex-filtered medium pressure Hg lamp (400 Watt, λ_max_ = 366 nm) cooled to room temperature was employed [43]. Continuous reagent delivery was ensured by a standard commercially available syringe pump (for details see Supporting Information File 1). Reactivity evaluation studies were performed utilizing a 2 mL FEP loop, for the gram-scale production of glycosylated building blocks a 5 mL FEP loop was used. This particular photochemical set up (Figure 2a) allows for several reaction parameters to be studied for later high scale synthesis of the glycosylated building blocks **8**–**13** while using only small amounts of reagents for optimization. As thiol–ene addition is strongly concentration dependent [26], similar concentrations for thioglycosides **2**–**7** during TEC are required for a valid comparison. Due to reagent solubility, a concentration of 0.1 M could be only realized by premixing all reagents before injection. In this case it is important to notice that no background reactivity could be measured when performing the reaction without irradiation under similar flow conditions.

Integration of the HPLC UV-signals at 254 nm was used to establish residence time versus conversion plots (Figure 2). The plots showed close to complete conversion within 30 min residence time and 1.5 equiv of thioglycoside β-Glc(OAc)_4_-SH **2** (95%) or β-Gal(OAc)_4_-SH **3** (94%) (Figure 2; Glc and Gal). Similar reactivities were also observed for monosaccharides **4**–**6**, supporting the substrate scope of this approach and its suitability to access a library of differently glycosylated building blocks. Referring to larger thio-substrate β-Lac(OAc)_7_-SH **7** a diminished reactivity with the same previously mentioned reaction conditions was determined (Figure 2b; Lac).

With optimized reaction conditions in place, large amounts of glycosylated building blocks are required to support solid phase oligomer synthesis. Large scale production of glycosylated building blocks **8**, **9**, **10** and **13** relied on the previously established conditions (30 min; 1.5 equiv thioglycoside; 0.1 M). Although the reactivity of aminoglycosides **5** and **6** is in the same range as that of glycosides **2**–**4**, we chose a higher excess of thiol component (2 equiv) for the production of glycosylated building blocks **11** and **12**, resulting in >95% conversion and an easy purification of the reaction mixture. Using this process, gram quantities of glycosylated building blocks **8**–**13** were obtained in 70–89% isolated yield after purification (Table 1).

**Table 1:** Overview of the preparation of glycosylated building block and used upscale conditions.

Building block	Thiol component	Equiv	Residence time	Conversion	Yield

β-GlcS_DDS **8**	β-Glc(OAc)_4_-SH **2**	1.5	30 min	95%	84%
β-GalS_DDS **9**	β-Gal(OAc)_4_-SH **3**	1.5	30 min	94%	81%
β-RhaS_DDS **10**	β-Rha(OAc)_3_-SH **4**	1.5	30 min	95%	89%
β-GlcNAcS_DDS **11**	β-GlcNAc(OAc)_3_-SH **5**	2	30 min	>95%	81%
β-GalNAcS_DDS **12**	β-GalNAc(OAc)_3_-SH **6**	2	30 min	>95%	85%
β-LacS_DDS **13**	β-Lac(OAc)_7_-SH **7**	1.5	30 min	84%	70%

**Figure 3:**
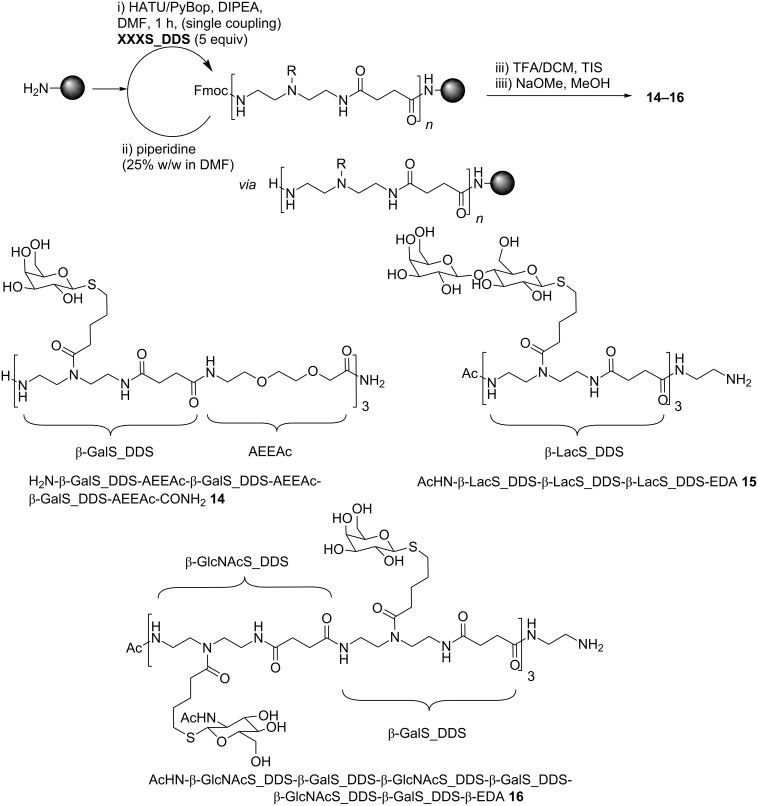
Solid phase coupling procedure and the obtained homo- and heteromultivalent glycoligands **14**–**16**.

With the isolated and characterized glyco-building blocks **8**–**13** obtained via TEC in flow, we then assembled three different glycooligomers **14**–**16** (Figure 3) to show the potential of the building block approach for the straightforward synthesis of a variety of differently glycosylated structures. The oligomer synthesis is based on standard peptide synthesis protocols and amide formation via activation of the building blocks’ free carboxy group, coupling to the solid support followed by deprotection of the amino group (Figure 3). This allows us to synthesize chemically defined oligomers with full control over the monomer sequence [15–16,18,26] using differently functionalized and spacer building blocks. Due to the use of fully functionalized building blocks the desired product can be obtained directly after cleavage from the resin and after overall deprotection.

In order to be suitable for solid-phase synthesis, the building blocks have to be soluble in DMF or NMP, permanent protective groups have to be stable towards piperidine exposure and the activated species should selectively react with primary amines without prior decomposition. Indeed, all glyco-building blocks described here, fulfil these criteria and can be applied for sequential coupling under PyBop or HATU activation and Fmoc deprotection with 25% piperidine in DMF on solid support (Figure 3). Final deprotection from the resin was performed with TFA/DCM mixtures, followed by acetyl deprotection in solution under Zemplén conditions [44]. Although the glycosylated building blocks **9**–**13** suffer from steric hindrance and have a relatively high molecular weight in comparison to amino acids, they show remarkably good coupling efficiency during amide bond formation on solid support. Glycooligomer **14**, combining three ß-GalS_DDS and three amino-diethoxyacetic acid (AEEAc) building blocks in alternating sequence, was accessible in high yields (81%) and purity (94% determined via integration of the HPLC signal at 214 nm) by referring only to single coupling (5 equiv) for one hour using PyBop activation (see Supporting Information File 1). After diethyl ether precipitation no further purification of compound **14** was necessary. This structure represents an example for a combination of glycosylated and non-glycosylated building blocks that allows for the variation of the density, number and spacing of sugar ligands along the scaffold. In order to test whether the building block approach is also suitable for the direct introduction of larger sugar ligands such as the Lac disaccharide, we synthesized structure **15** presenting three Lac ligands placed right next to each other in a short oligomer chain. Indeed, depending on the building blocks applied and oligomer structures targeted, different activating reagents are required. Glycooligomer **15** introducing Lac groups was obtained as highly pure material after solid-phase synthesis using HATU activation instead of PyBop followed by HPLC purification. Another important advantage of the presented building block approach is the straightforward access to so-called heteromultivalent glycooligomers. Here different sugar ligands are presented at different positions along the oligomer chain. To date, such heteromultivalent systems are mainly obtained by functionalization with mixtures of sugar ligands that do not allow for a precise positioning of the different sugar ligands [9,45]. Alternatively, a polymer-analogue strategy is required where different functional groups are placed along a polymer chain that allow for orthogonal conjugation strategies introducing the different sugars sequentially [37,46]. Our approach simply relies on choosing from our differently glycosylated building blocks that are introduced in the desired pattern by automated solid-phase synthesis. As a proof of principle we synthesized glycoligand **16** as a multivalent scaffold that presents two different monosaccharides. β-GlcNAc and β-Gal are exposed in alternating fashion with an overall oligomer length of six building blocks and a molecular weight of 3000 Da. Similar to glycooligomer **15** this structure was obtained as highly pure material after solid-phase synthesis using HATU activation and HPLC purification.

## Conclusion

In this article we reported on the synthesis of glycosylated building blocks via photochemical thiol–ene chemistry in a continuous-flow reactor using five different monosaccharides and one disaccharide. We showed that this flow set up provides excellent conversion rates with several substrates. All monosaccharides were shown to react under the same conditions with equivalent conversion rates, whereas the peracetylated β-thio-lactose as a disaccharide showed slightly diminished reactivity. Additionally, these small scale reactions were transferred into the gram-scale production of six different glycosylated building blocks.

These carbohydrate presenting building blocks were then applied for solid-phase synthesis resulting in three monodisperse, sequence-defined glycooligomers with different glycosylation patterns. The building-block approach for the synthesis of glycooligomers thus allows for the control of the ligand positioning as well as the straightforward introduction of defined sequences of different types of ligands. Ongoing studies focus on the synthesis of a larger set of different glycooligo/polymers and the evaluation of their binding properties as sugar mimetics. For example, homomultivalent oligomer **14** and analogues are characterized for their interactions with asialoglycoprotein receptors, while heteromultivalent oligomer **16** represents a potential mimic of poly(lacNAc), which is known to be an important naturally-occurring galectin binder.

## Experimental

**General TEC procedure.** A photoreactor was set up using 5 mL (for optimization reactions 2 mL) loop of FEP tubing around a Pyrex and a medium pressure Hg lamp [26,43]. A recirculating chiller (Huber Unistat 360, filled with spectroscopically pure water as coolant) was used to maintain the reactor at a temperature of 25–30 °C (for further details see Supporting Information File 1). Using a syringe pump (Harvard PHD2000), a solution of DDS **1** (1.0 equiv), acetyl-protected thioglycosidesglycoside **2**–**7** (1.5–2.0 equiv) and acetic acid (3 equiv) in degassed methanol was injected into the photoreactor. The entire reactor output was collected and evaporated under reduced pressure to afford the crude material.

**β-GlcS_DDS (8):** A solution of acetyl protected β-thioglucose **2** (1.35 g; 3.69 mmol, 1.5 equiv) and DDS **1** (1.25 g; 2.46 mmol, 1.0 equiv) in MeOH (24 mL) and AcOH (0.42 mL) (residence time, 30 min; flow rate 167 μL min^−1^) was reacted under photochemical conditions according to the general TEC procedure. The reactor outcome was concentrated and purified via silica chromatography (DCM/MeOH + 1% AcOH 15:1) giving compound **8** (1.8 g; 84%). The analytical data is in accordance with published data [26].

**β-GalS_DDS (9):** A solution of acetyl protected β-thiogalactose **3** (1.35 g; 3.69 mmol, 1.5 equiv) and DDS **1** (1.25 g; 2.46 mmol, 1.0 equiv) in MeOH (24 mL) and AcOH (0.42 mL) (residence time, 30 min; flow rate 167 μL min^−1^) was reacted under photochemical conditions according to the general TEC procedure. The reactor outcome was concentrated and purified via silica chromatography (DCM/MeOH + 1% AcOH 15:1) giving compound **9** (1.74 g; 81%). IR (film) ν: 2945, 1748, 1225 cm^−1^; [α]_D_^20^ −32.19 (*c* 1, MeOH); ^1^H NMR (400 MHz, CDCl_3_) 7.76 (d, *J* = 7.5, 2H), 7.58 (d, *J* = 7.4, 2H), 7.39 (t, *J* = 7.4, 2H), 7.30 (t, *J* = 7.4, 2H), 7.07–6.83 (m, 1H), 5.86–5.64 (m, 1H), 5.47–5.35 (m, 1H), 5.19 (t, *J* = 9.8, 1H), 5.10–4.97 (m, 1H), 4.65–4.27 (m, 3H), 4.25–3.80 (m, 4H), 3.62–3.18 (m, 8H), 2.73–2.53 (m, 4H), 2.52–2.29 (m, 4H), 2.12 (s, 3H), 2.05–1.99 (m, 6H), 1.97 (s, 3H), 1.76–1.50 (m, 4H); ^13^C NMR (100 MHz, CDCl_3_) (mixture of rotamers) 175.0, 173.1, 172.9, 170.7, 170.4, 170.2, 169.9, 156.9, 143.9, 143.9, 141.4, 141.4, 127.9, 127.2, 125.2, 125.1, 120.2, 120.2, 84.1, 74.5, 72.0, 67.5, 67.5, 67.0, 61.5, 48.5, 48.3, 47.3, 45.9, 40.1, 39.9, 39.3, 38.6, 32.6, 32.4, 30.9, 29.8, 29.4, 29.4, 29.3, 24.5, 24.5, 20.9, 20.8, 20.8, 20.7; HRMS (ESI) *m*/*z*: [M + Na]^+^ calcd for C_42_H_53_N_3_O_15_SNa, 894.3095; found, 894.3096; RP-HPLC analysis, 5% to 95% MeCN in 10 min, retention time = 8.1 min.

**β-RhaS_DDS (10):** A solution of acetyl protected L-β-thiorhamnose **4** (1.13 g; 3.69 mmol, 1.5 equiv) and DDS **1** (1.25 g; 2.46 mmol, 1.0 equiv) in MeOH (24 mL) and AcOH (0.42 mL) (residence time, 30 min; flow rate 167 μL min^−1^) was reacted under photochemical conditions according to the general TEC procedure. The reactor outcome was concentrated and purified via silica chromatography (DCM/MeOH + 1% AcOH 17:1) giving compound **10** (1.78 g; 89%). IR (film) ν: 2940, 1745, 1630, 1224 cm^−1^; [α]_D_^20^ −5.83 (*c* 1, MeOH); ^1^H NMR (400 MHz, CDCl_3_) 7.74 (d, *J* = 7.5, 2H), 7.57 (d, *J* = 7.4, 2H), 7.38 (t, *J* = 7.3, 2H), 7.29 (t, *J* = 7.6, 2H), 6.96 (br s, 1H), 5.86–5.65 (m, 1H), 5.30 (dd, *J* = 3.3, 1.5, 1H), 5.22–5.16 (m, 1H), 5.14 (s, 1H), 5.07 (t, *J* = 9.8, 1H), 4.46–4.26 (m, 2H), 4.24–4.12 (m, 2H), 3.67–3.09 (m, 8H), 2.82–2.23 (m, 8H), 2.17–2.09 (m, 3H), 2.04 (s, 3H), 1.97 (s, 3H), 1.73–1.56 (m, 4H), 1.19 (d, *J* 6.2, 3H); ^13^C NMR (100 MHz, CDCl_3_) (mixture of rotamers) 175.3, 175.2, 174.9, 174.8, 173.1, 172.9, 170.4, 170.1, 170.1, 157.3, 156.9, 143.9, 143.9, 141.4, 141.4, 127.9, 127.2, 125.2, 125.1, 120.1, 120.1, 82.3, 71.8, 71.3, 69.7, 67.3, 67.0, 48.5, 48.2, 47.3, 46.2, 45.9, 40.1, 39.8, 39.2, 38.6, 32.6, 32.4, 31.1, 30.8, 30.8, 29.7, 29.4, 29.2, 29.1, 24.5, 24.4, 21.1, 20.9, 20.8, 17.5, 17.5;; HRMS (ESI) *m*/*z*: [M + Na]^+^ calcd for C_40_H_51_N_3_O_13_SNa, 836.3040; found, 836.3056; RP-HPLC analysis, 5% to 95% MeCN in 10 min, retention time = 7.8 min.

**β-GlcNAcS_DDS (11):** A solution of acetyl protected β-thioglucosamine **5** (0.72 g; 1.97 mmol, 2.0 equiv) and DDS **1** (0.5 g; 0.99 mmol, 1.0 equiv) in MeOH (10 mL) and AcOH (0.17 mL) (residence time, 30 min; flow rate 167 μL min^−1^) was reacted under photochemical conditions according to the general TEC procedure. The reactor outcome was concentrated and purified via silica chromatography (DCM/MeOH + 1% AcOH 10:1) giving compound **11** (0.70 g; 81%). IR (film) ν: 2940, 1744, 1654, 1229 cm^−1^; [α]_D_^20^ +25.65 (*c* 2, MeOH); ^1^H NMR (400 MHz, CDCl_3_) 7.77–7.71 (m, 2H), 7.57 (d, *J* = 7.3, 2H), 7.37 (t, *J* = 7.4, 2H), 7.33–7.25 (m, 2H), 5.21–4.99 (m, 2H), 4.64–4.50 (m, 1H), 4.41–3.99 (m, 6H), 3.72–3.56 (m, 1H), 3.51–3.17 (m, 8H), 2.76–2.52 (m, 4H), 2.50–2.28 (m, 4H), 2.02 (s, 3H), 2.00–1.95 (m, 6H), 1.89 (d, *J* = 9.1 Hz, 2H), 1.75–1.42 (m, 2H); ^13^C NMR (100 MHz, CDCl_3_) (mixture of rotamers) 174.8, 174.7, 173.2, 171.2, 171.2, 171.1, 170.1, 169.5, 169.5, 157.2, 157.0, 143.8, 141.3, 127.9, 127.2, 125.1, 120.1, 83.7, 83.7, 75.7, 74.1, 68.6, 68.6, 67.0, 66.9, 62.4, 53.0, 53.0, 48.2, 47.9, 47.2, 46.1, 45.8, 39.7, 38.5, 38.3, 32.3, 32.1, 30.9, 29.8, 29.3, 29.3, 28.3, 23.9, 23.8, 23.1, 20.9, 20.8, 20.7; HRMS (ESI) *m*/*z*: [M + Na]^+^ calcd for C_42_H_54_N_4_O_14_SNa, 893.3255; found, 893.3263; RP-HPLC analysis, 5% to 95% MeCN in 10 min, retention time = 7.1 min.

**β-GalNAcS_DDS (12):** A solution of acetyl protected β-thiogalactosamine **6** (0.72 g; 1.97 mmol, 2.0 equiv) and DDS **1** (0.5 g; 0.99 mmol, 1.0 equiv) in MeOH (10 mL) and AcOH (0.17 mL) (residence time, 30 min; flow rate 167 μL min^−1^) was reacted under photochemical conditions according to the general TEC procedure. The reactor outcome was concentrated and purified via silica chromatography (DCM/MeOH + 1% AcOH 10:1) giving compound **12** (0.73 g; 85%). IR (film) ν: 1746, 1655, 1236 cm^−1^; [α]_D_^20^ +145.42 (*c* 2, MeOH); ^1^H NMR (400 MHz, CDCl_3_) 7.77–7.69 (m, 2H), 7.56 (d, *J* = 7.3, 2H), 7.36 (t, *J* = 7.4, 2H), 7.32–7.23 (m, 2H), 5.39–5.25 (m, 1H), 5.14–5.01 (m, 1H), 4.57 (t, *J* = 10.5, 1H), 4.41–3.94 (m, 6H), 3.90–3.77 (m, 1H), 3.54–3.04 (m, 8H), 2.78–2.52 (m, 4H), 2.51–2.27 (m, 4H), 2.10 (s, 3H), 2.05–1.84 (m, 9H), 1.79–1.45 (m, 4H); ^13^C NMR (100 MHz, CDCl_3_) (mixture of rotamers) 175.2, 174.8, 174.7, 173.1, 173.0, 171.5, 171.3, 170.8, 170.7, 170.5, 170.5, 157.2, 143.8, 143.8, 141.3, 128.0, 127.2, 125.2, 125.1, 120.1, 84.2, 84.0, 74.3, 71.7, 71.6, 67.0, 66.9, 61.8, 61.8, 49.2, 48.2, 47.8, 47.3, 46.2, 45.8, 39.7, 38.5, 38.4, 32.3, 32.1, 30.7, 30.6, 29.7, 29.6, 29.4, 28.4, 28.3, 23.9, 23.8, 23.2, 23.2, 20.8, 20.7; HRMS (ESI) *m*/*z*: [M + Na]^+^ calcd for C_42_H_54_N_4_O_14_SNa, 893.3255; found, 893.3247; RP-HPLC analysis, 5% to 95% MeCN in 10 min, retention time = 7.1 min.

**β-LacS_DDS (13):** A solution of acetyl protected β-thiolactose **7** (0.79 g; 1.18 mmol, 1.5 equiv) and DDS **1** (0.4 g; 0.79 mmol, 1.0 equiv) in MeOH (7.9 mL) and AcOH (0.17 mL) (residence time, 30 min; flow rate 167 μL min^−1^) was reacted under photochemical conditions according to the general TEC procedure. The reactor outcome was concentrated and purified *via* silica chromatography (DCM/MeOH + 1% AcOH 15:1) giving compound **13** (0.65 g; 70%). IR (film) ν: 1750, 1230, 1051 cm^−1^; [α]_D_^20^ −35.00 (*c* 2, MeOH); ^1^H NMR (400 MHz, CDCl_3_) 7.72 (d, *J* = 7.5, 2H), 7.55 (d, *J* = 7.5, 2H), 7.37 (t, *J* = 7.3, 2H), 7.29–7.25 (m, 2H), 5.31 (s, 1H), 5.16 (dt, *J* = 9.2, 3.1, 1H), 5.06 (t, *J* = 7.9, 1H), 4.96–4.85 (m, 2H), 4.47–4.04 (m, 9H), 3.87–3.71 (m, 2H), 3.55 (br s, 1H), 3.45–3.29 (m, 8H), 2.63–2.54 (m, 4H), 2.44–2.29 (m, 2H), 2.34–2.27 (m, 2H); 2.11 (s, 3H), 2.06–2.05 (m, 3H), 2.02–1.98 (m, 12H), 1.93 (s, 3H), 1.67–1.52 (m, 4H); ^13^C NMR (100 MHz, CDCl_3_) (mixture of rotamers) 174.9, 174.7, 172.8, 172.7, 170.6, 170.5, 170.3, 170.3, 170.1, 170.0, 169.8, 169.8, 169.7, 169.7, 169.6, 169.1, 169.1, 157.0, 156.7, 143.8, 143.7, 141.3, 141.2, 127.8, 127.7, 127.1, 125.0, 125.0, 120.0, 120.0, 101.0, 83.2, 76.1, 73.8, 70.9, 70.6, 70.3, 69.1, 66.9, 66.8, 66.6, 62.2, 62.1, 60.8, 60.8, 48.3, 47.9, 47.1, 46.00, 45.6, 39.9, 38.9, 38.4, 32.4, 32.2, 30.7, 29.7, 29.6, 29.5, 29.2, 29.1, 24.2, 20.8, 20.7, 20.7, 20.6, 20.4; HRMS (ESI) *m*/*z*: [M + Na]^+^ calcd for C_54_H_69_N_3_O_23_SNa, 1182.3940; found, 1182.3956; RP-HPLC analysis, 5% to 95% MeCN in 10 min, retention time = 9.3 min.

**H_2_N-β-GalS_DDS-AEEAc- β-GalS_DDS-AEEAc- β-GalS_DDS-AEEAc-CONH_2_ (14):** Compound **14** (30 mg, 16.2 μmol) was obtained as white hygroscopic powder after cleavage from the resin, precipitation into diethyl ether and deactylation with a yield of 81%. MALDI–TOF–MS: [M + Na]^+^ calcd for C_75_H_135_N_13_O_33_S_3_Na, 1864.83 (monoisotopic); found, 1864.63; RP-HPLC analysis 5% to 95% MeCN in 10 min, retention time = 3.9 min.

**AcHN-β-LacS_DDS-β-LacS_DDS-β-LacS_DDS-EDA (15):** Acetyl protected compound **15** was cleaved from the resin and precipitated into diethyl ether. The crude material was purified via preparative RP-HPLC (5 to 95% MeCN in 30 min) and freezed-dried. After final deactylation compound **15** (11 mg, 5.6 μmol) was obtained as white hygroscopic powder with 28% yield. MALDI–TOF–MS: [M + H]^+^ calcd for C_79_H_140_N_11_O_4_0S_3,_ 1978.84 (monoisotopic); found, 1979.06; [M + Na]^+^ calcd for C_79_H_139_N_11_O_4_0S_3_Na, 2000.82 (monoisotopic); found, 2000.88; [M + K]^+^ calcd for C_79_H_139_N_11_O_4_0S_3_K, 2016.80 (monoisotopic); found, 2017.01; RP-HPLC analysis 5% to 50% MeCN in 30 min, retention time = 5.9 min.

**AcHN-β-GlcNAcS_DDS-β-GalS_DDS-β-GlcNAcS_DDS-β-GalS_DDS-β-GlcNAcS_DDS-β-GalS_DDS-β-EDA (16)** Acetyl protected compound **16** was cleaved from the resin and precipitated into diethyl ether. After deactylation in solution, the crude material was purified via preparative RP-HPLC (5 to 50% MeCN in 30 min) and freezed-dried. Compound **16** (12.6 mg, 4.2 μmol) was obtained as white hygroscopic powder with 21% yield. MALDI–TOF–MS: [M + Na]^+^ calcd for C_124_H_217_N_23_O_49_S_6_Na, 3027.34 (monoisotopic); found, 3027.71; RP-HPLC analysis 5% to 50% MeCN in 30 min, retention time = 7.8 min.

## Supporting Information

File 1 Further experimental procedures, characterization data and spectra.
