# Supplementary material for: Synthesis of homo- and heteromultivalent carbohydrate-functionalized oligo(amidoamines) using novel glyco-building blocks
Source: Beilstein J Org Chem. 2013 Nov 7;9:2395–403. doi: 10.3762/bjoc.9.276 (PMC3869284; doi:10.3762/bjoc.9.276)
Supplement: File 1 — Further experimental procedures, characterization data and spectra. [file Beilstein_J_Org_Chem-09-2395-s001.pdf]

Supporting Information

for

Synthesis of homo- and heteromultivalent carbohydrate-functionalized oligo(amidoamines) using novel glyco-building blocks

Felix Wojcik, Sinaida Lel, Alexander G. O'Brien, Peter H. Seeberger* and Laura Hartmann*

Address: Department of Biomolecular Systems, Max Planck Institute of Colloids and Interfaces, Research Campus Golm, 14424 Potsdam, Germany, and Institute for Chemistry and Biochemistry, Freie Universität Berlin, Arnimallee 22, 14195 Berlin, Germany

Email: Peter.Seeberger@mpikg.mpg.de; Laura.Hartmann@mpikg.mpg.de

* Corresponding author

Further experimental procedures, characterization data and spectra

Contents

1. Experimental procedures S2–S7

1.1 General experimental details. S2

1.2 Construction and configuration of the continuous flow reactors. S3

1.3 Experimental procedures and analytical data. S4–S7

2 NMR Spectra S8–S15

3 RP-HPLC analysis S16–S17

4 MALDI-spectra S18–S19

5 References S20

1 Experimental procedures

1.1 General experimental details

Commercial grade reagents and solvents were used without further purification. ^{1}H NMR and ^{13}C NMR spectra were measured with a Varian 400-MR and a Varian 600-MR spectrometer. The proton signal of residual, non-deuterated solvent (δ 7.26 ppm for $CHCl_3$) was used as an internal reference for ^{1}H NMR spectra. For ^{13}C NMR spectra, the chemical shifts are reported relative to the δ 77.36 ppm resonance of $CDCl_3$. Coupling constants (J) are reported in Hertz (Hz). The following abbreviations are used to indicate the multiplicities: s, singlet; d, doublet; t, triplet; m, multiplet. Infrared spectra were recorded as thin films on a Perkin Elmer Spectrum 100 FTIR spectrophotometer. Analytical thin-layer chromatography (TLC) was performed on Kieselgel 60 F254 aluminium plates precoated with a 0.20 mm thickness of silica gel. The TLC plates were visualized with UV light and by staining with basic potassium permanganate solution. Column chromatography was performed using Kieselgel 60 (230–400 mesh). The solid-support resin was purchased from Rapp Polymers, coupling agents from Novabiochem and Fmoc-AEEAc-OH (2-[2-(Fmoc-amino)ethoxy]ethoxy)acetic acid) from Iris Biotech. Solid phase reactions were performed on an automated Activotec P11 Peptide Synthesizer. Analytical reverse phase HPLC (RP-HPLC) was performed on Agilent 1200 HPLC System using an Agilent Eclipse (4.6 × 100 mm) C18 column at a flow rate of 1 mL/min. The purity was determined by integration of the UV-signal. MALDI–TOF–MS spectra were recorded on a Bruker Autoflex Speed II in Reflector Mode and 2,5-dihydroxybenzoic acid (DHB) as matrix.

1.2 Construction and configuration of photoflow reactor for TEC [1,2].

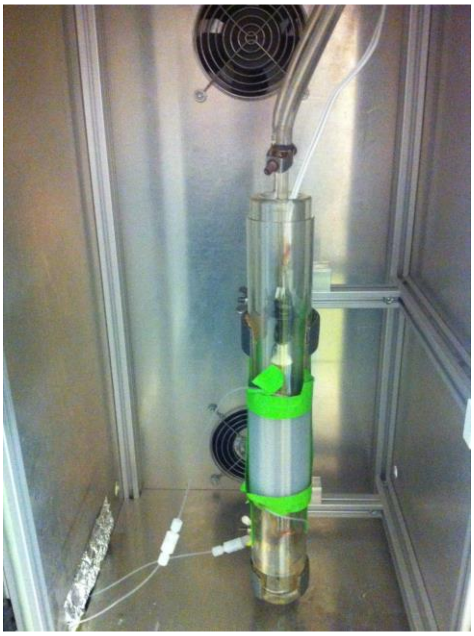

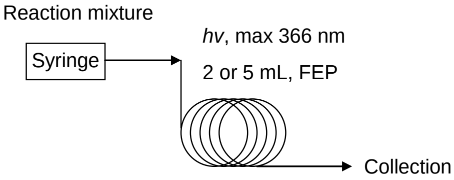

The flow reactor setup is described in a similar manner as in [2]. The setup consisted of a Harvard PHD2000 syringe pump and multiple loops of FEP tubing (fluorinated ethylene polymer from IDEX Health & Science 1520, natural color, outside diameter (OD) 1/16 in and inside diameter (ID) 0.030 in) wrapped tightly around a Pyrex filter (inner diameter 4.5 cm and wall thickness 0.2 cm), surrounding the quartz immersion well cooled by a recirculating cryostat (Huber Unistat 360), a medium pressure Hg lamp (UV 450 immersion lamp 5 in. arc, radial lead, 7825-34 from Ace Glass), a power supply for photochemical lamp (7830 from Ace Glass) and a collection flask. The temperature in the tube during the reaction is estimated to range from 25 to 30 °C, based on temperature measurements taken between the cooling jacket and the tube. For safety reasons, the lamp was places inside an aluminium box for blocking UV irradiation. Two fans were installed for additional cooling.

1. 3 Experimental procedures and analytical data.

1.3.1 Thioglycosides

β-Glc(OAc)$_4$-SH (2*R*,3*R*,4*S*,5*R*,6*S*)-2-(acetoxymethyl)-6-mercaptotetrahydro-2*H*-pyran-3,4,5-triyl triacetate (2)

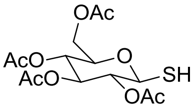

2

Prepared according to [3]. δ_H (400 MHz, $CDCl_3$) 5.17 (t, *J* = 9.3 Hz, 1H), 5.08 (t , *J* = 9.7 Hz, 1H), 4.95 (t, *J* = 9.5 Hz, 1H), 4.53 (d, *J* 9.7 Hz, 1H), 4.22 (1H, dd, *J* = 12.5 Hz, 4.8 Hz), 4.10 (1H, dd, *J* = 12.5 Hz, 2.3 Hz), 3.70 (1H, ddd, *J* 10.0, 4.8, 2.3), 2.29 (3H, s), 2.06 (3H, s), 2.06 (3H, s), 1.99 (3H, s), 1.99 (3H, s); δ_C (100 MHz, $CDCl_3$) 170.6, 170.0, 169.5, 169.3, 78.7, 76.3, 73.5, 73.5, 68.1, 61.9, 20.7, 20. 7, 20.5, 20.5.

β-Gal(OAc)$_4$-SH (2*R*,3*S*,4*S*,5*R*,6*S*)-2-(acetoxymethyl)-6-mercaptotetrahydro-2*H*-pyran-3,4,5-triyl triacetate (3)

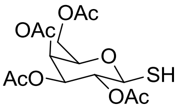

3

Prepared according to [3].δ_H (400 MHz, $CDCl_3$) 5.41 (dd, *J* = 3.4, 1.1 Hz, 1H), 5.16 (t, *J* = 9.9 Hz, 1H), 5.00 (dd, *J* = 10.1, 3.4 Hz, 1H), 4.52 (t, *J* = 9.8 Hz, 1H), 4.11 (d, *J* = 6.6 Hz, 2H), 3.93 (dt, *J* = 6.6, 1.2 Hz, 1H), 2.35 (d, *J* = 9.9 Hz, 1H), 2.14 (s, 3H), 2.07 (s, 3H), 2.03 (s, 3H), 1.96 (s, 3H); δ_C (100 MHz, $CDCl_3$) 170.4, 170.2, 170.0, 169.9, 79.3, 75.1, 71.7, 71.0, 67.4, 61.6, 20.9, 20.8, 20.8, 20.7.

1 Experimental procedures

β-Rha(OAc)$_3$-SH (2*R*,3*R*,4*R*,5*S*,6*S*)-2-mercapto-6-methyltetrahydro-2*H*-pyran-3,4,5-triyl triacetate (4)

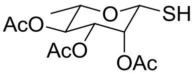

4

Prepared according to [4].δ_H (400 MHz, CDCl$_3$) 5.47 (d, *J* = 6.9 Hz, 1H), 5.34 – 5.28 (m, 2H), 5.14 – 5.06 (m, 1H), 2.23 (d, *J* = 6.9 Hz, 1H), 2.15 (s, 3H), 2.06 (s, 3H), 1.99 (s, 3H), 1.23 (d, *J* = 6.2 Hz, 3H); δ_C (100 MHz, CDCl$_3$) 170.1, 170.1, 170.0, 76.9, 72.5, 71.2, 68.7, 67.8, 21.0, 20.9, 20.8, 17.4.

β-GlcNAc(OAc)$_3$-SH (2*R*,3*S*,4*R*,5*R*,6*S*)-5-acetamido-2-(acetoxymethyl)-6-mercapto-tetrahydro-2*H*-pyran-3,4-diyl diacetate (5)

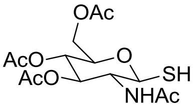

5

Prepared according to [3]. δ_H (400 MHz, CDCl$_3$) 5.84 (d, *J* = 9.4 Hz, 1H), 5.14 – 5.03 (m, 2H), 4.60 (dd, *J* = 10.0, 9.4 Hz, 1H), 4.22 (dd, *J* = 12.4, 4.9 Hz, 1H), 4.16 – 4.03 (m, 2H), 3.69 (ddd, *J* = 9.8, 4.8, 2.4 Hz, 1H), 2.54 (d, *J* = 9.3 Hz, 1H), 2.08 (s, 3H), 2.03 – 2.00 (m, 6H), 1.97 (s, 3H); δ_C 171.3, 170.8, 170.6, 169.3, 80.4, 76.4, 73.7, 68.3, 62.3, 57.0, 23.4, 20.9, 20.8, 20.7.

β-GalNAc(OAc)$_3$-SH (2*R*,3*R*,4*R*,5*R*,6*S*)-5-acetamido-2-(acetoxymethyl)-6-mercapto-tetrahydro-2*H*-pyran-3,4-diyl diacetate (6)

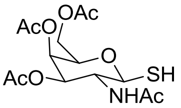

6

Prepared according to [5]. δ_H (400 MHz, CDCl$_3$) 5.73 (d, *J* = 9.5 Hz, 1H), 5.37 (dd, *J* = 3.3, 1.0 Hz, 1H), 5.06 (dd, *J* = 10.9, 3.4 Hz, 1H), 4.61 (t, *J* = 9.4 Hz, 1H), 4.25 (dd, *J* = 20.6, 9.8 Hz, 1H), 4.11 (d, *J* = 6.6 Hz, 2H), 3.92 (dt, *J* = 6.6, 1.1 Hz, 1H), 2.60 (d, *J* = 9.1 Hz, 1H), 2.16

(s, 3H), 2.08 – 1.94 (m, 9H); δ_C 170.9, 170.8, 170.6, 170.4, 80.8, 75.0, 71.1, 67.0, 61.8, 53.3, 23.5, 20.9, 20.9, 20.8.

β-Lac(OAc)$_7$-SH (2*R*,3*S*,4*S*,5*R*,6*S*)-2-(acetoxymethyl)-6-(((2*R*,3*R*,4*S*,5*R*,6*S*)-4,5-diacetoxy-2-(acetoxymethyl)-6-mercaptotetrahydro-2*H*-pyran-3-yl)oxy)tetrahydro-2*H*-pyran-3,4,5-triyl triacetate (7)

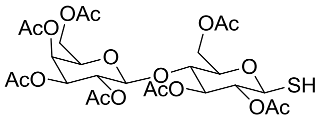

7

Prepared according to [4] δ_H (400 MHz, $CDCl_3$) 5.32 (dd, *J* = 3.5, 1.1 Hz, 1H), 5.15 (t, *J* = 9.2 Hz, 1H), 5.07 (dd, *J* = 10.4, 7.9 Hz, 1H), 4.93 (dd, *J* = 10.4, 3.5 Hz, 1H), 4.85 (t, *J* = 9.6 Hz, 1H), 4.57 – 4.38 (m, 3H), 4.14 – 4.00 (m, 1H), 3.89 – 3.82 (m, 3H), 3.82 – 3.74 (m, 1H), 3.66 – 3.56 (m, 1H), 2.23 (d, *J* = 9.6 Hz, 1H), 2.15 – 2.08 (m, 6H), 2.07 – 2.00 (m, 12H), 1.93 (s, 3H); δ_C δ 170.9, 170.1, 170.1, 169.9, 169.8, 169.6, 169.4, 168.9, 100.9, 78.3, 77.2, 77.0, 76.9, 76.6, 75.9, 73.7, 73.3, 70.8, 70.6, 68.9, 66.5, 62.1, 60.7, 60.2, 20.8, 20.7, 20.6, 20.5, 20.4, 20.3, 14.0.

1.3.2 TEC residence time optimization

General TEC pilot optimization procedure. In order to access the reactivity of different thioglycosides during TEC, a photoreactor was set up using 2 mL (loop of FEP tubing around a Pyrex and a medium pressure Hg lamp [1,2] (see 1.2 construction and configuration of photoflow reactor for TEC). A solution of DDS **1** (1.0 equiv), acetyl protected thioglycosides **2**–**7** (1.5–2.0 equiv) and acetic acid (3 equiv) (total volume 2 mL, corresponding to the volume of the reactor) in degassed methanol was injected into the photoreactor. Both before and after the injected sample a plug (0.3 mL) of Argon was injected. The sample and Ar plugs were then pushed through the reactor with pure methanol. The entire reactor output was collected and evaporated under reduced pressure to afford the crude material.

1.3.3 Solid-phase synthesis

In a similar manner as described in [2] all solid phase reactions were performed on an automated standard peptide synthesizer at 0.02 mmol scale according to the following general

solid phase protocols. Tentagel S RAM resin (loading 0.24 mmol/g) and ethylenediamine preloaded Tentagel S Trityl resin (loading 0.20 mmol/g) were used as solid supports, and were swollen twice for 15 min in DCM before starting the initial Fmoc-deprotection or coupling protocols.

Coupling/Fmoc-deprotection protocol. In a similar manner as described in [2] 5 equiv of the glyco-building blocks **8–13** and O-(7-Azabenzotriazol-1-yl)-*N,N,N',N'*-tetramethyluronium hexafluorophosphate (HATU) (37 mg, 98 μmol, 4.9 equiv) were placed as powder in the amino acid vial and placed in the peptide synthesizer. The solids were dissolved in DMF (1 mL) under nitrogen stream. Then *N*,*N*-diisopropylethylamine (DIEA) (0.2 mL of a 1 M solution in DMF, 10 equiv) was added. Preactivation was carried out for 3 min in the amino acid vial before the solution was transferred to the resin. The resin with the coupling solution was shaken carefully for 1 hour, the reaction vessel was emptied and washed with DMF.

Fmoc deprotection was performed using 25% piperidine in DMF for 5 min and checked by UV monitoring for the fluorenyl piperidine adduct at 301 nm. This step was repeated until the deprotection was complete.

Final cleavage from solid support. In a similar manner as described in [2] the final cleavage was performed by adding the cleavage cocktail (50% DCM, 47.5% TFA and 2.5% TIS 1 mL/50 mg resin) to the resin and allowing it to react for 60 min using the Tentagel S RAM resin. The resin cleavage for the EDA-Trityl Tentagel resin was carried out with 10% TFA and 90% DCM for 20 min and was performed twice for complete cleavage of the oligomers. The cleavage solution was filtered and poured onto ice cold diethyl ether. The white precipitate was collected by centrifugation and washed twice with diethyl ether to give acetyl protected compound **14**–**16**.

Deprotection of the acetyl groups. Fully protected oligomers **14**–**16** were dissolved in MeOH (3 mL). Then NaOMe (0.8 mL; 10 mg/mL in MeOH) was added slowly. The reaction was monitored via RP-HPLC. After complete deprotection the reaction mixture was neutralized using a cation exchange resin (H^+-form), filtered and evaporated under reduced pressure. The product was dissolved in water and lyophilized to give the final deprotected products. For oligomer **16** acetic acid was used for neutralization, then the crude mixture was purified by preparative RP-HPLC.

2. NMR spectra

^{1}H NMR (400 MHz, $CDCl_3$)

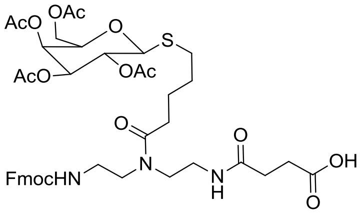

9

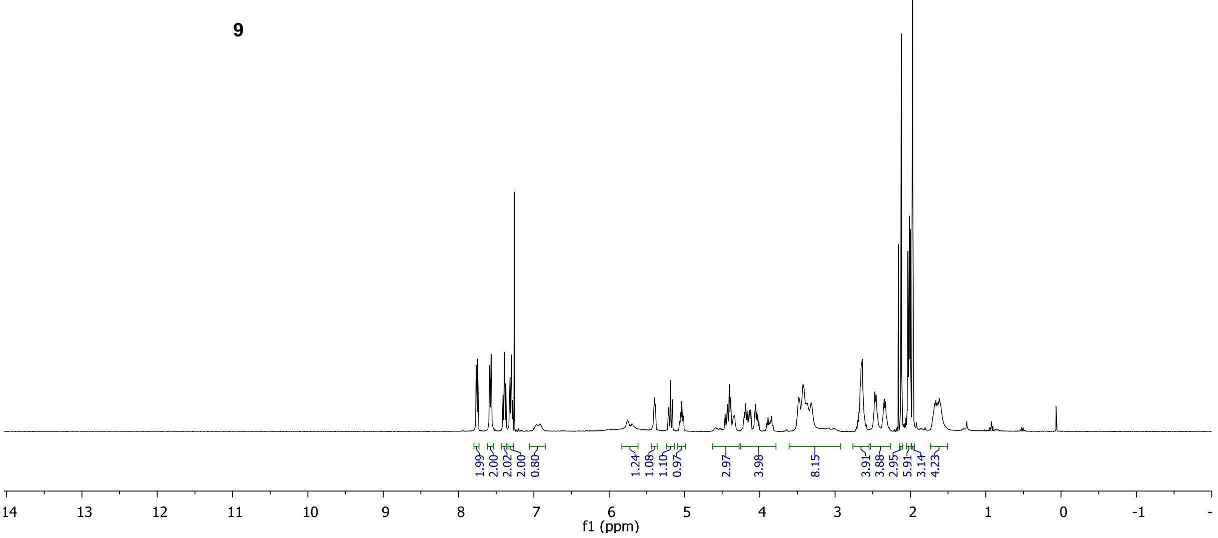

^{13}C NMR (100 MHz, $CDCl_3$)

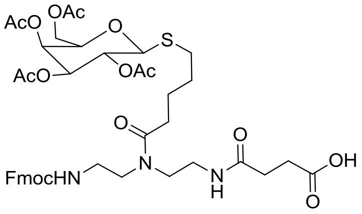

9

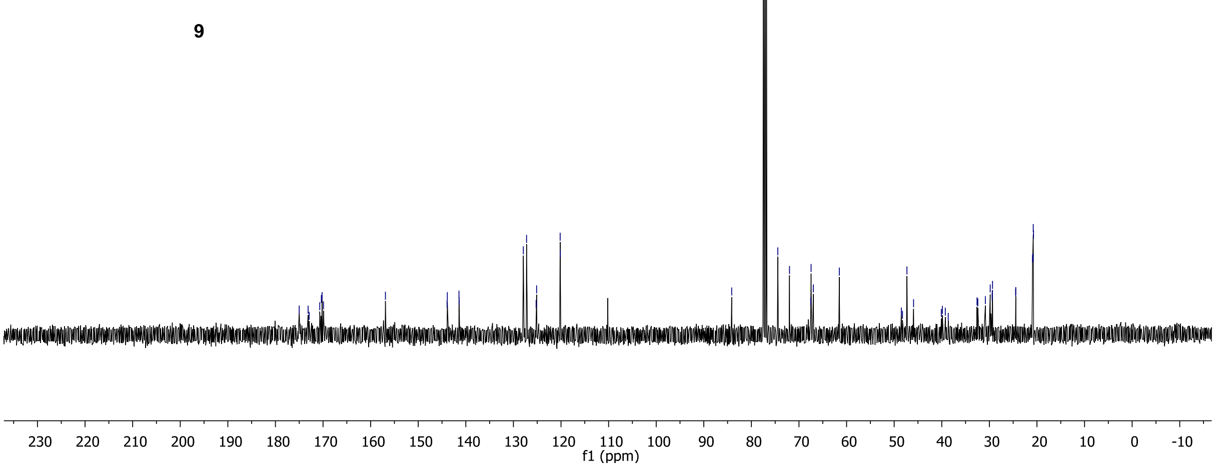

2. NMR spectra

HSQC ($CDCl_3$) compound **9**

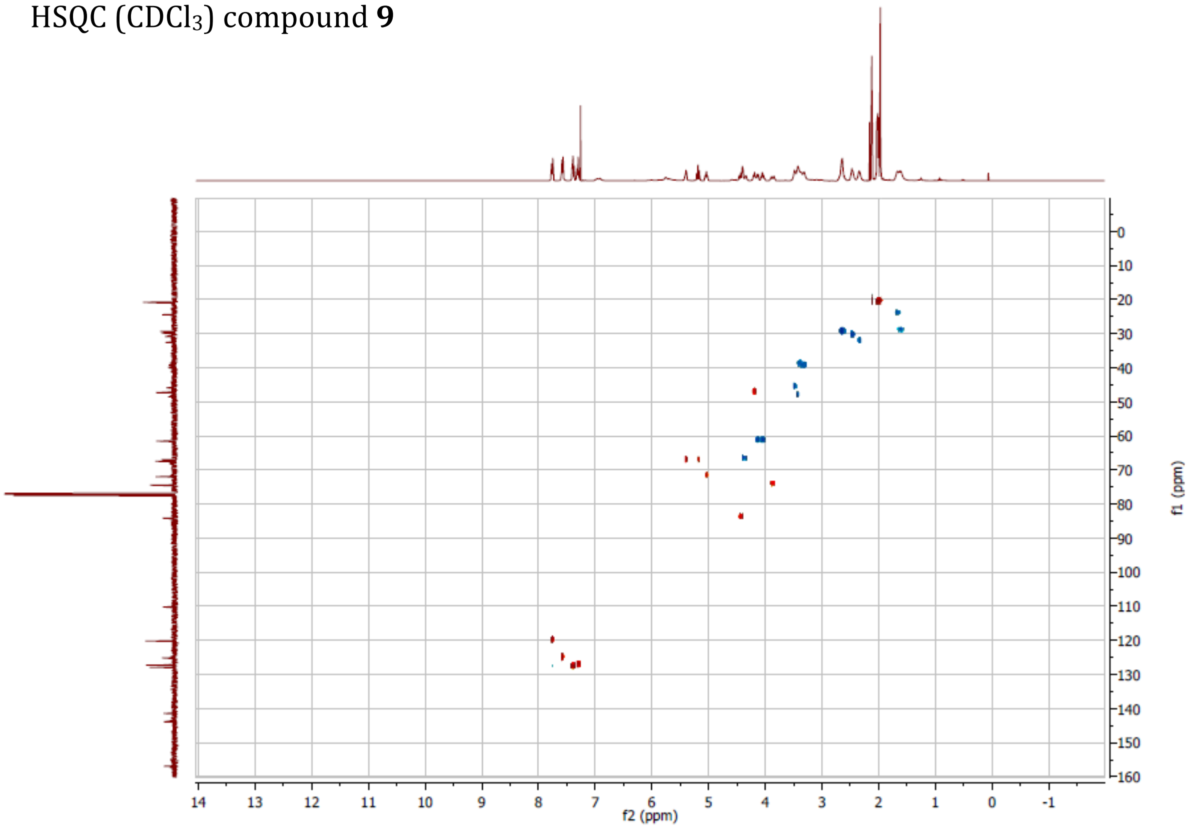

^{1}H NMR (400 MHz, $CDCl_3$)

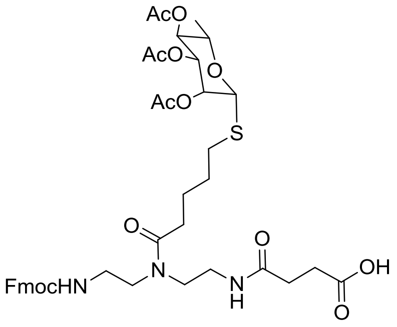

10

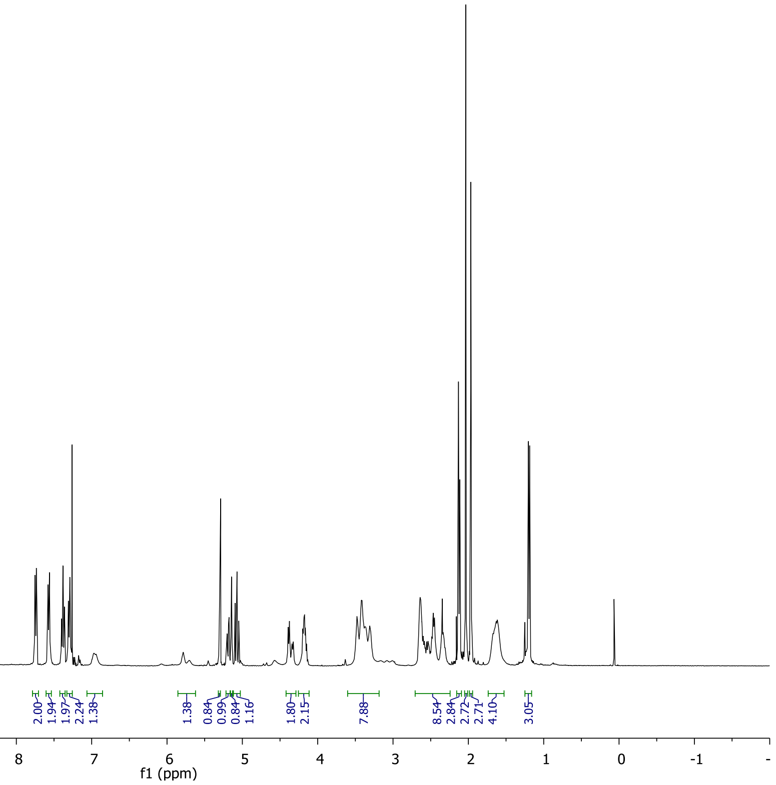

2. NMR spectra

^{13}C NMR (100 MHz, $CDCl_3$)

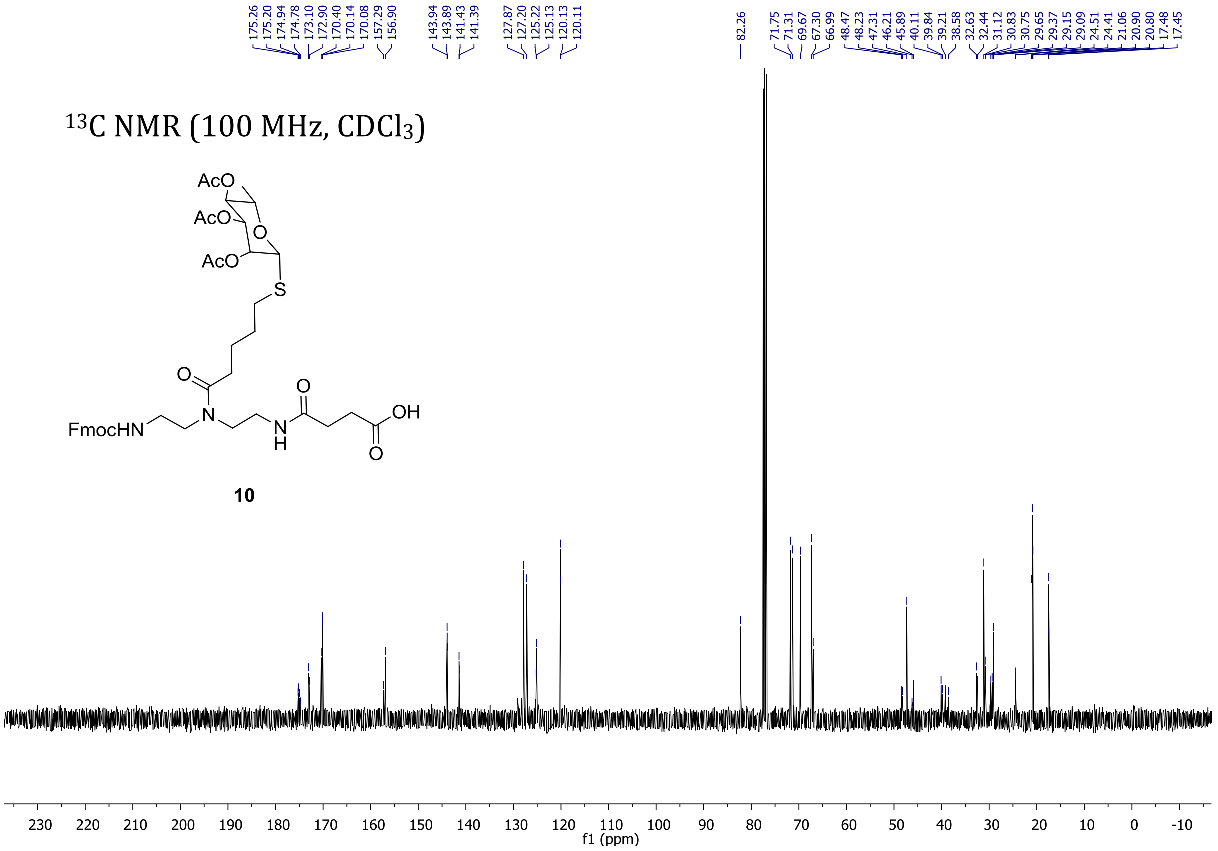

HSQC ($CDCl_3$) compound **10**

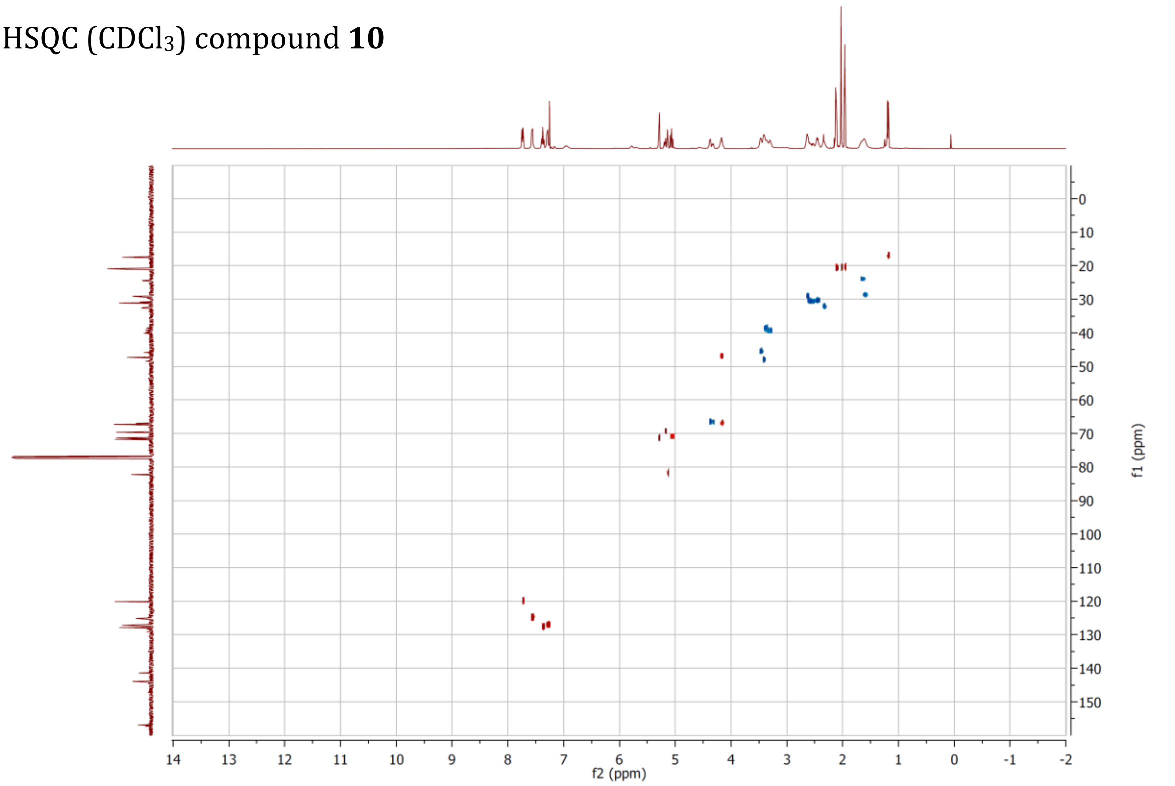

2. NMR spectra

^{1}H NMR (400 MHz, $CDCl_3$)

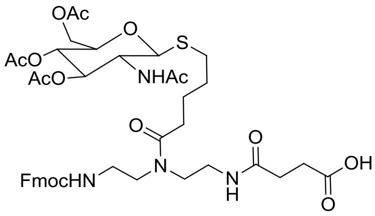

11

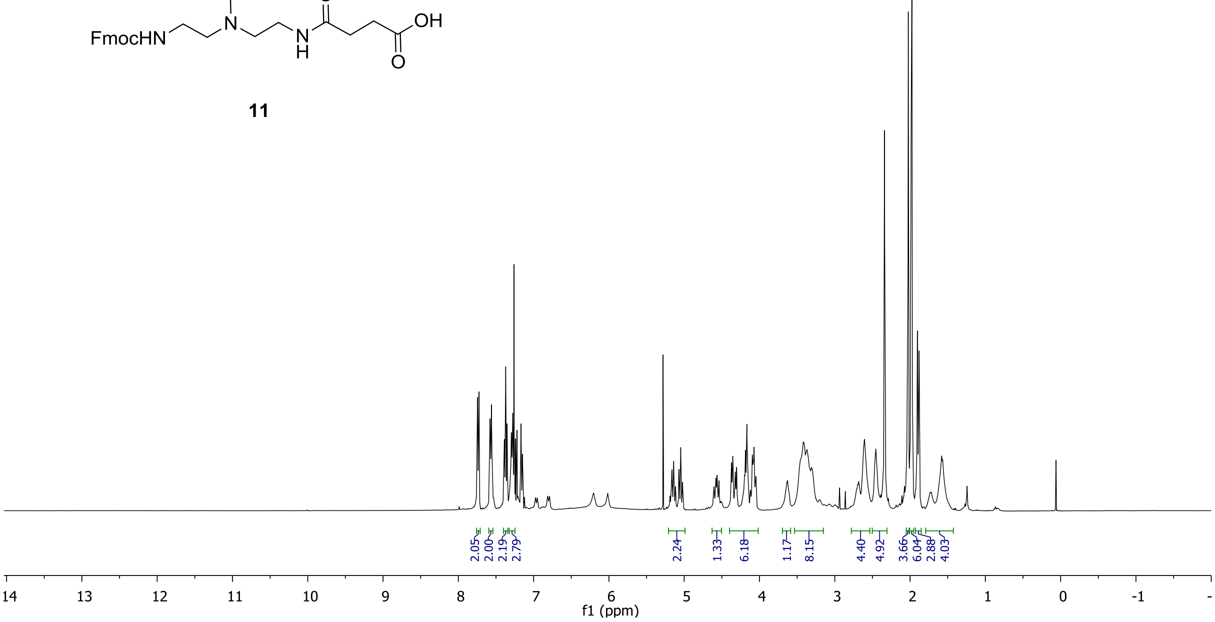

^{13}C NMR (100 MHz, $CDCl_3$)

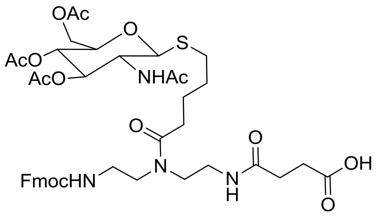

11

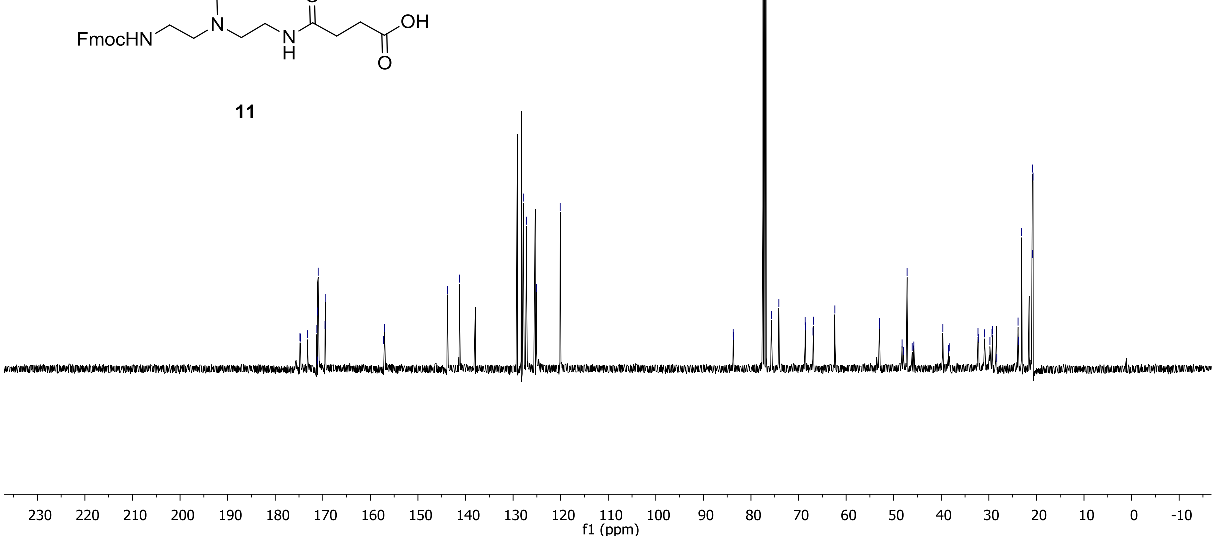

2. NMR spectra

HSQC ($CDCl_3$) compound **11**

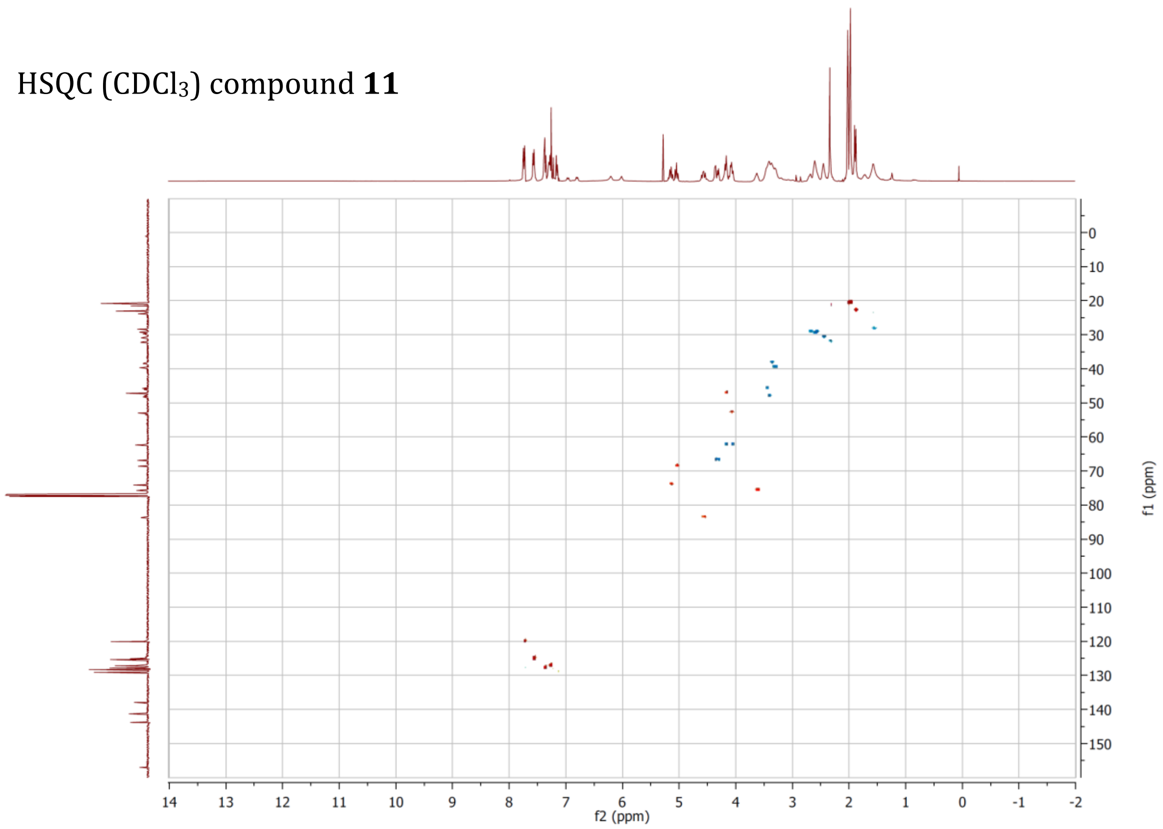

^1H NMR (400 MHz, $CDCl_3$)

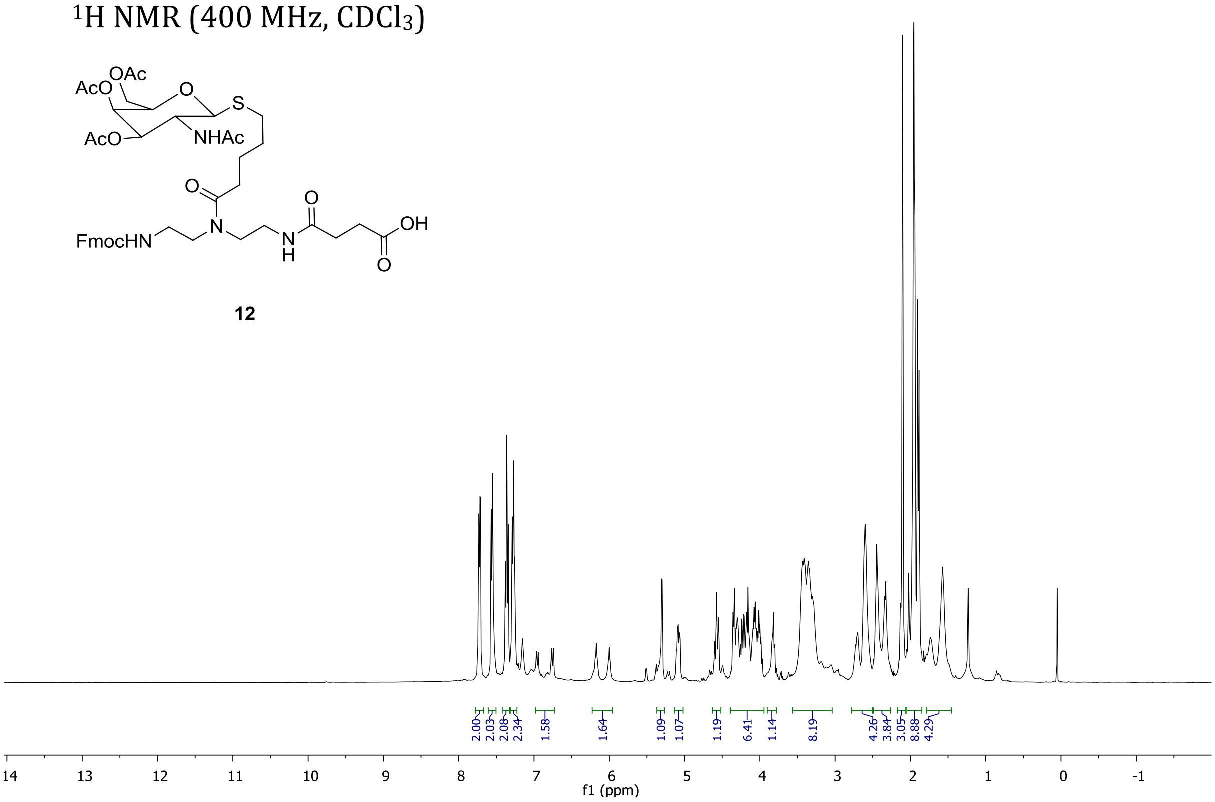

2. NMR spectra

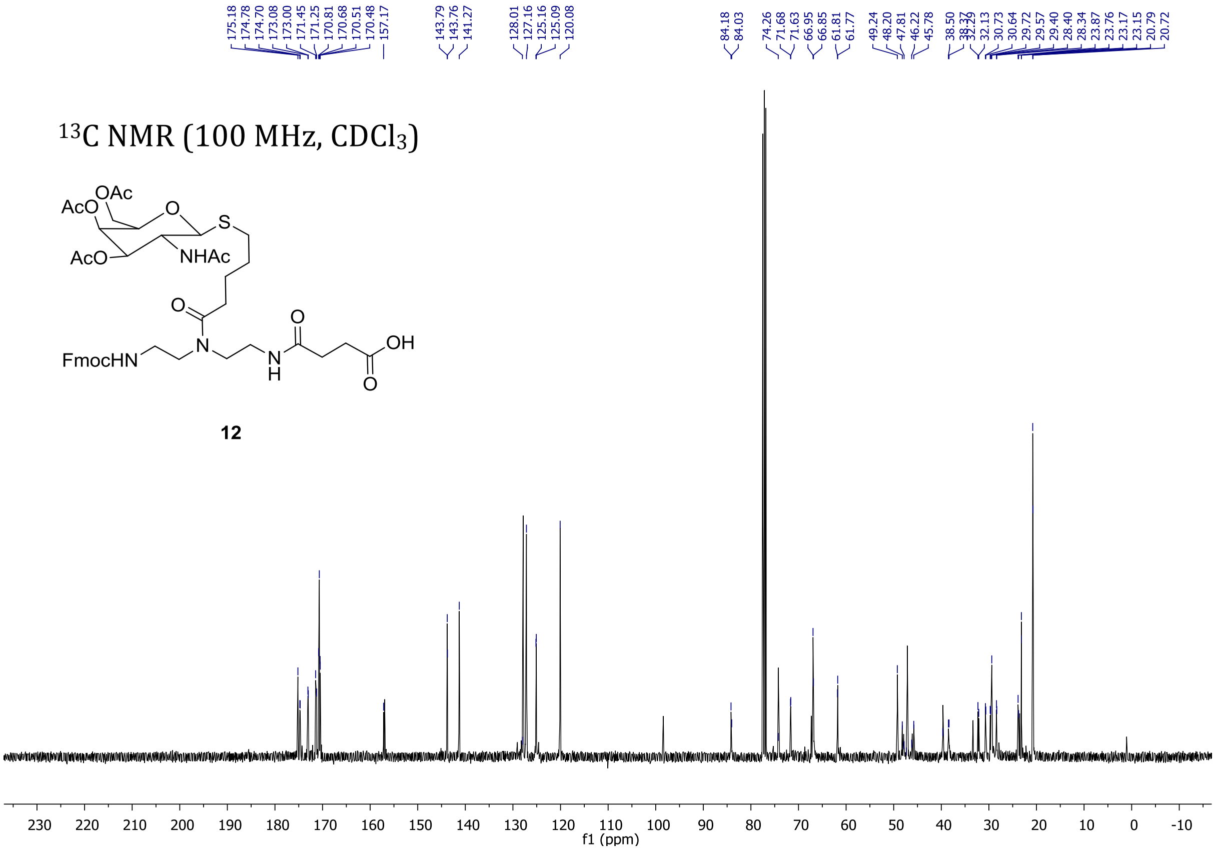

^{13}C NMR (100 MHz, CDCl$_3$)

12

HSQC (CDCl$_3$) compound **12**

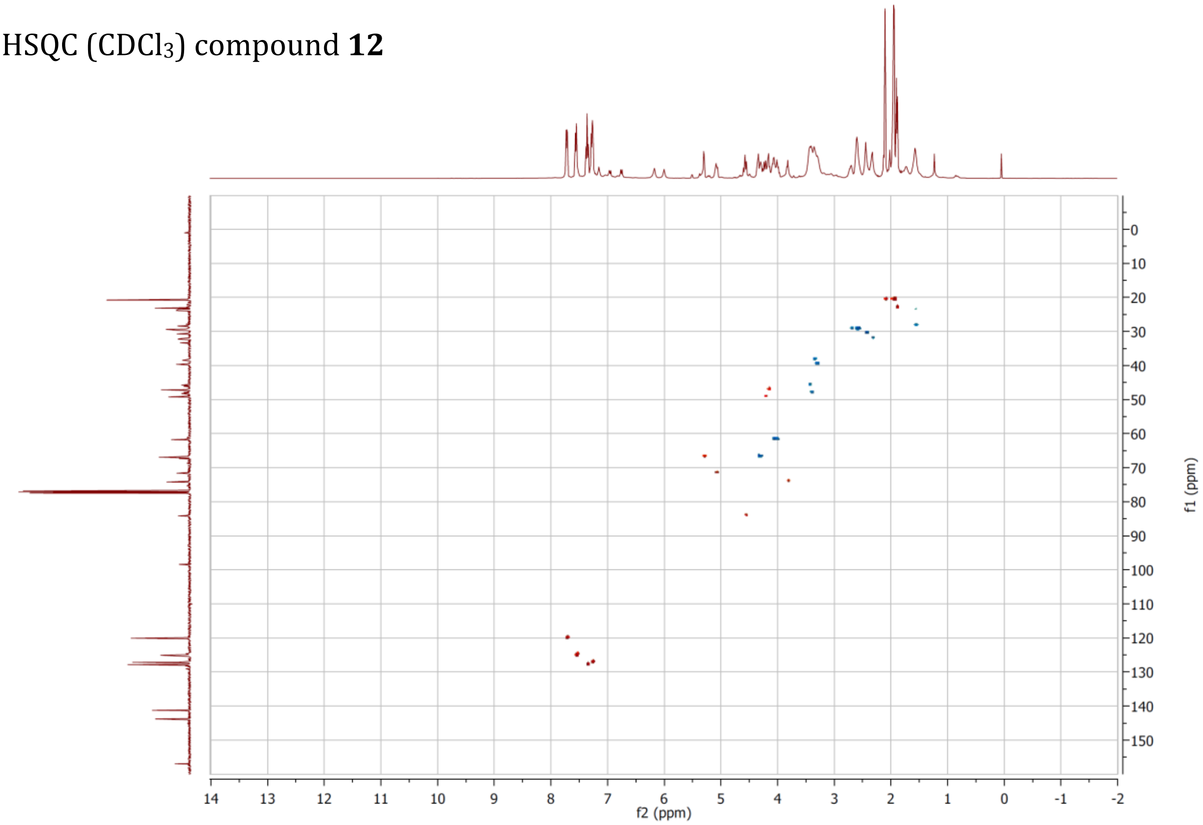

2. NMR spectra

^{1}H NMR (400 MHz, $CDCl_3$)

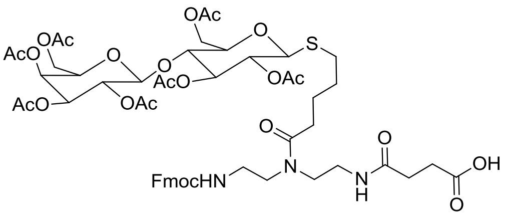

13

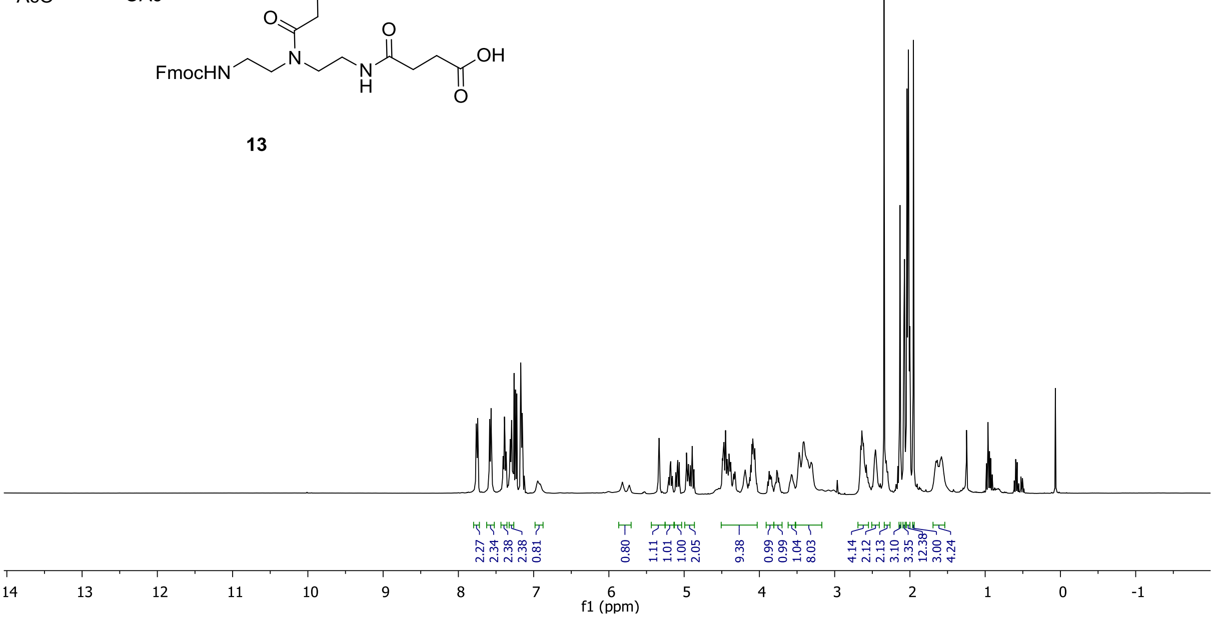

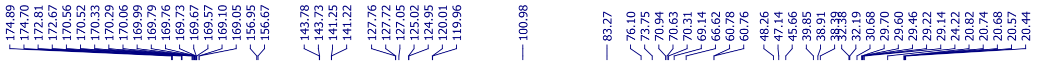

^{13}C NMR (100 MHz, $CDCl_3$)

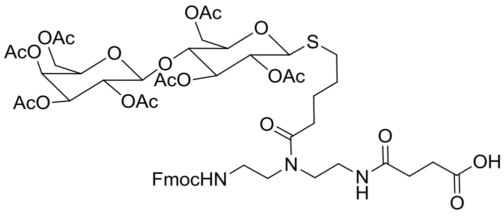

13

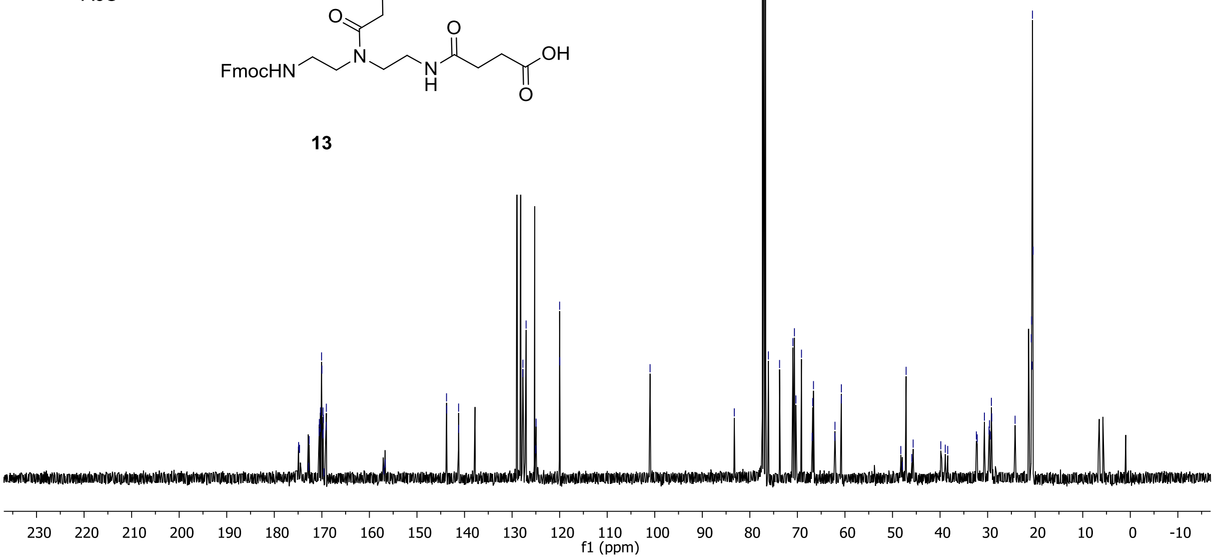

HSQC ($CDCl_3$) compound **13**

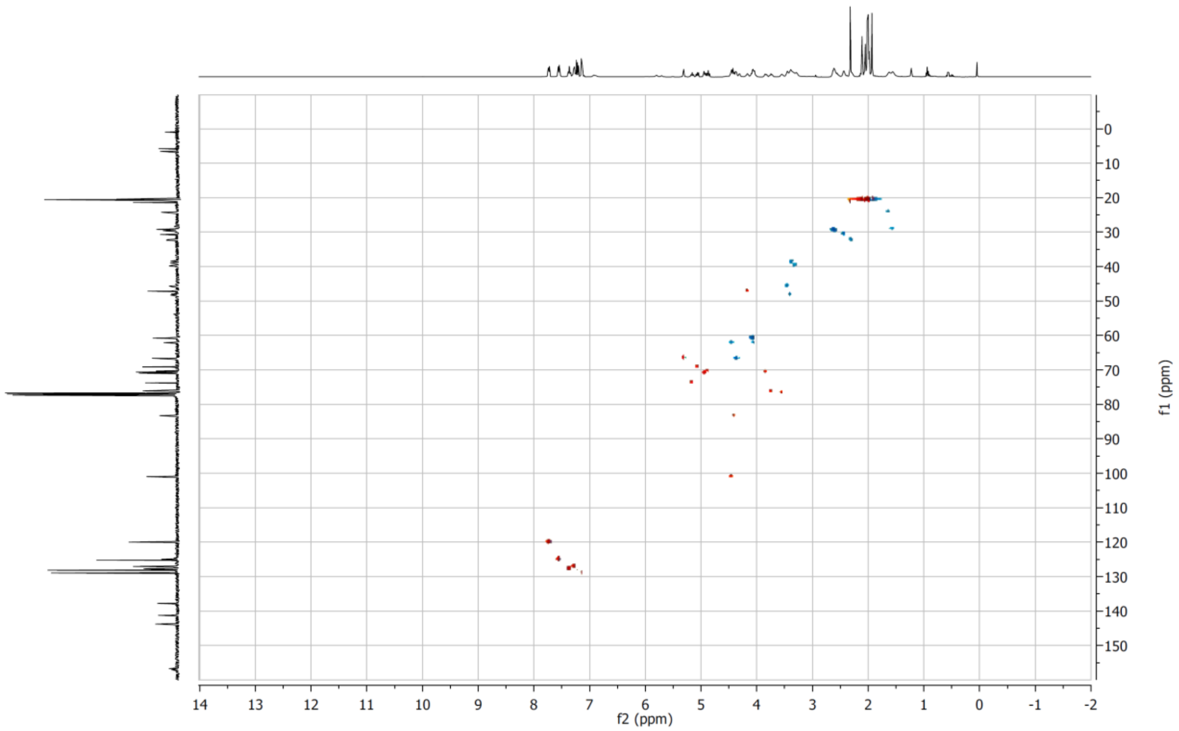

3. RP-HPLC-analysis

RP-HPLC of compound **14**

Eluent A: H_2O + 0.1% TFA
Eluent B: MeCN + 0.1% TFA
Gradient: Linear 5-95% B in 10 min

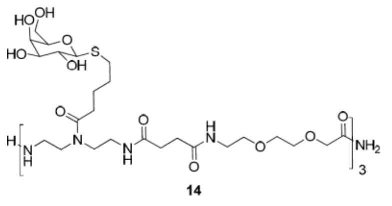

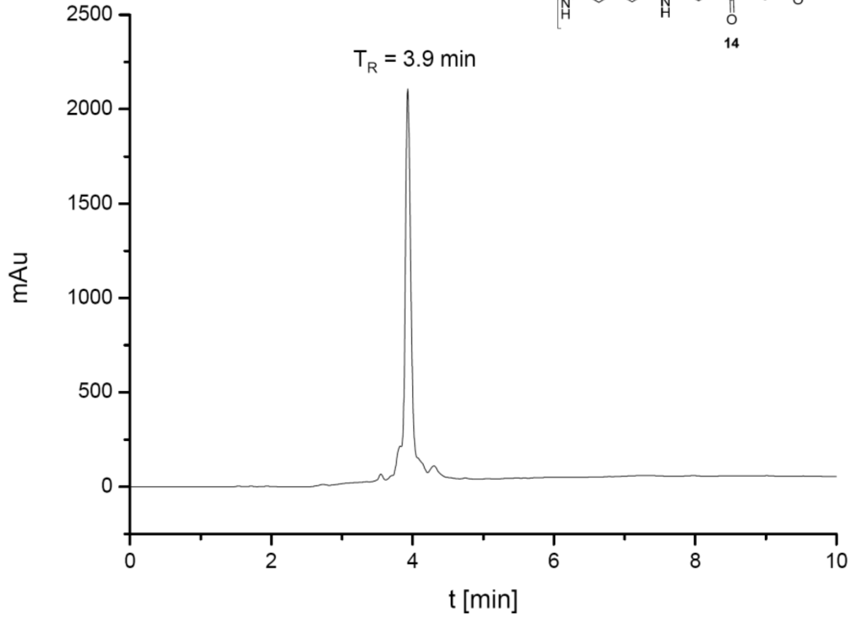

RP-HPLC of compound **15**

Eluent A: H_2O + 0.1% TFA
Eluent B: MeCN + 0.1% TFA
Gradient: Linear 5-50% B in 30 min

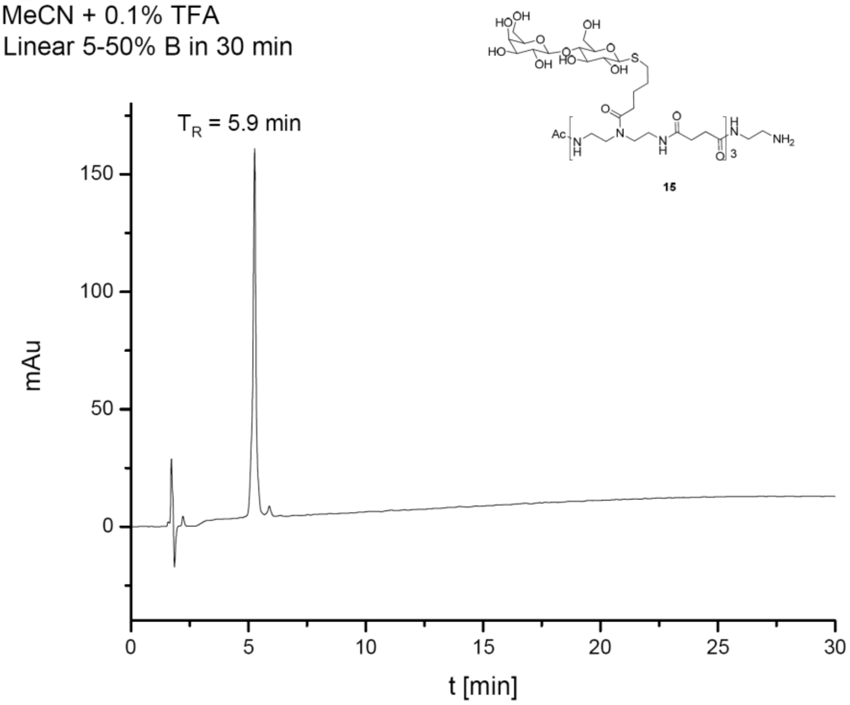

3. RP-HPLC-analysis

RP-HPLC of compound **16**

Eluent A: H_2O + 0.1% TFA
Eluent B: MeCN + 0.1% TFA
Gradient: Linear 5-50% B in 30 min

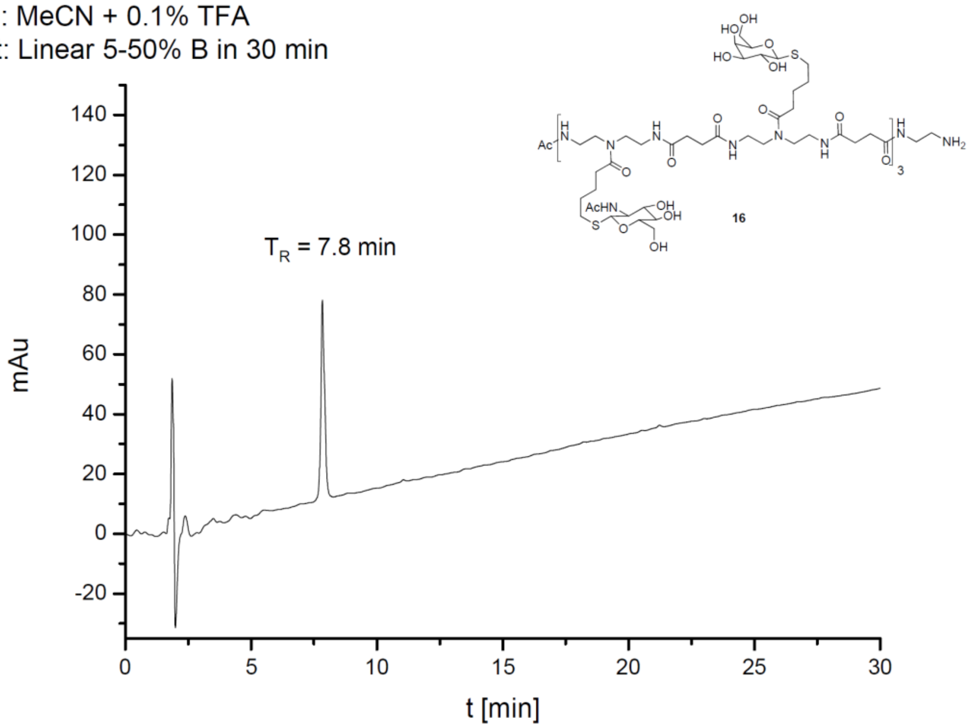

4. MALDI spectra

Reflector Mode

Matrix: DHB (2,5-dihydroxybenzoic acid)

Compound **14**

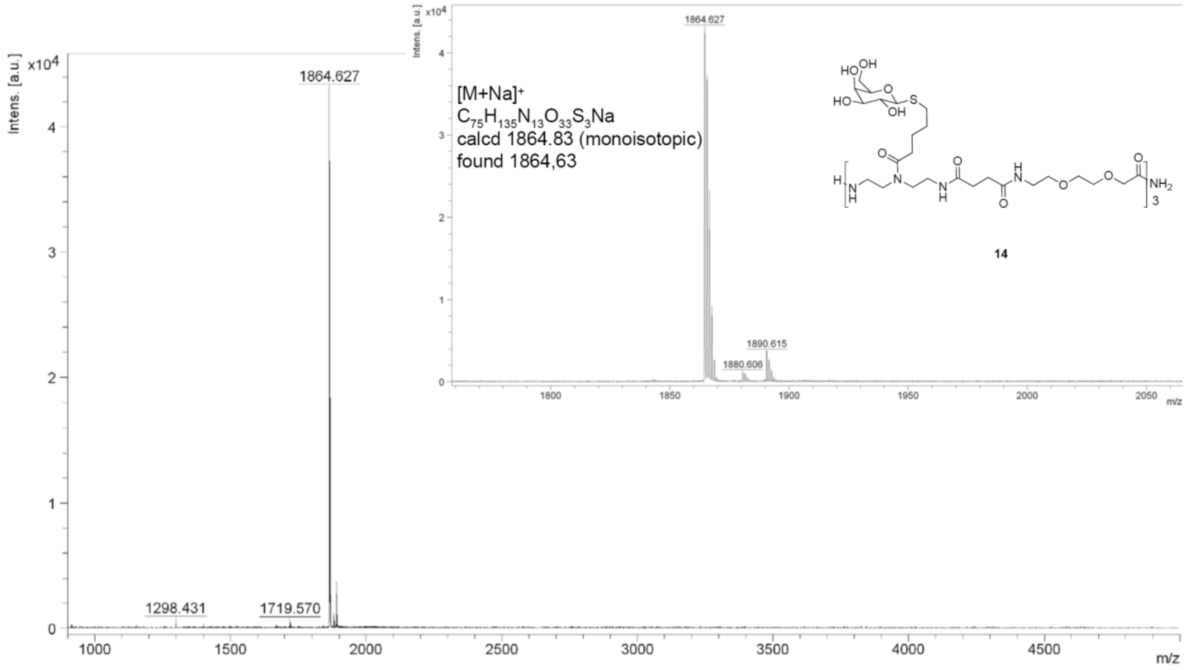

Reflector Mode

Matrix: DHB (2,5-dihydroxybenzoic acid)

Compound **15**

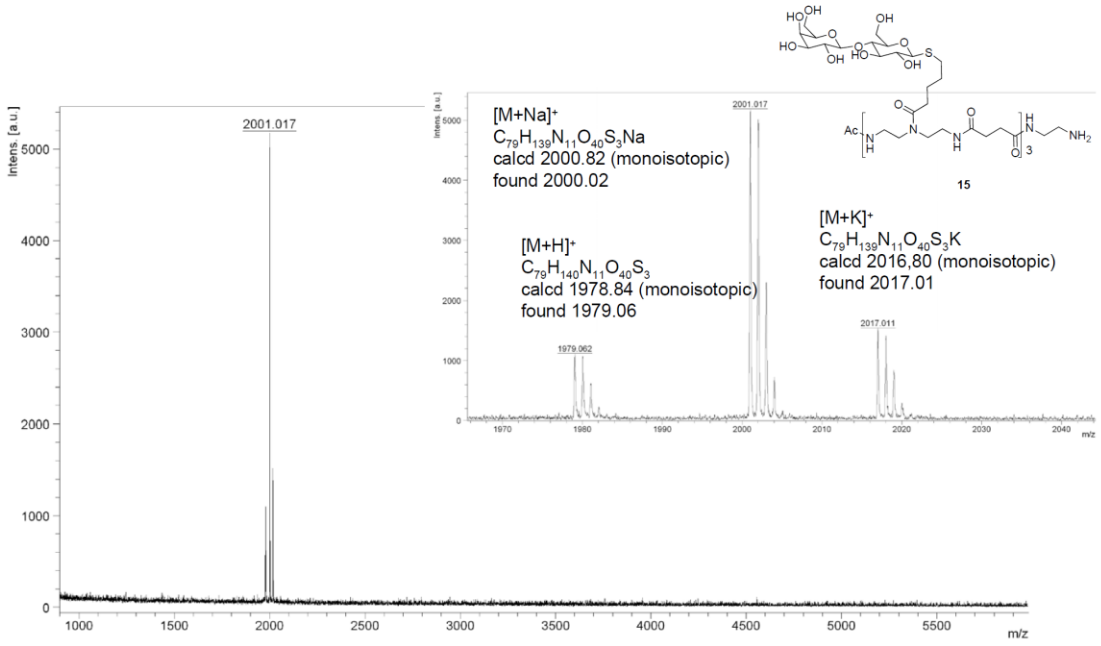

4. MALDI spectra

Reflector Mode

Matrix: DHB (2,5-dihydroxybenzoic acid)

Compound **16**

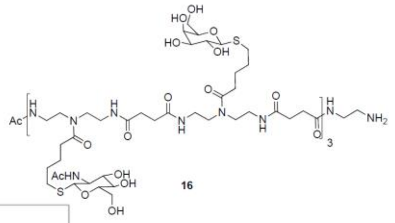

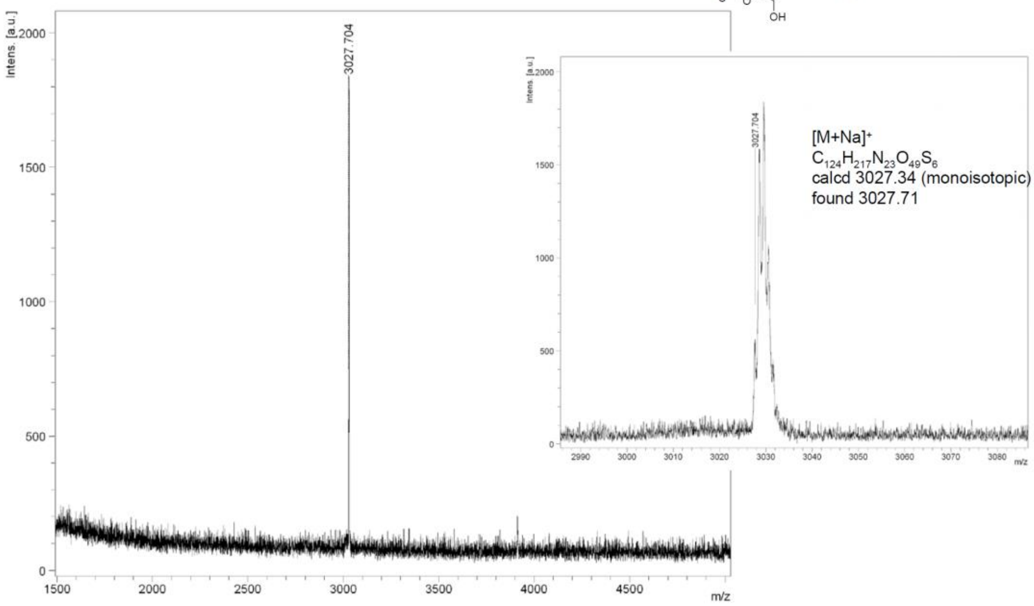

References

[1] Hook, B. D. A.; Dohle, W.; Hirst, P. R.; Pickworth, M.; Berry, M. B.; Booker-Milburn, K. I. *J. Org. Chem.* **2005**, *70*, 7558-7564.

[2] Wojcik, F.; O'Brien, A. G.; Gotze, S.; Seeberger, P. H.; Hartmann, L. *Chem.–Eur. J.* **2013**, *19*, 3090-3098.

[3] N. Floyd, B. Vijayakrishnan, A. R. Koeppe, B. G. Davis, *Angew. Chem., Int. Ed.* **2009**, *48*, 7798-7802.

[4] M. Jana, A. K. Misra, *J. Org. Chem.* **2013**, *78*, 2680–2686.

[5] C. S. Rye, S. G. Withers, *Carbohydr. Res.* **2004**, *339*, 699–703.
